# Variations of five eIF4E genes across cassava accessions exhibiting tolerant and susceptible responses to cassava brown streak disease

**DOI:** 10.1371/journal.pone.0181998

**Published:** 2017-08-03

**Authors:** Shanshan Shi, Xiuchun Zhang, M. Alejandra Mandel, Peng Zhang, Yuliang Zhang, Morag Ferguson, Teddy Amuge, Steve Rounsley, Zhixin Liu, Zhongguo Xiong

**Affiliations:** 1 Institute of Tropical biology and biotechnology, Chinese Academy of Tropical Agricultural Sciences, Haikou, China; 2 School of Plant Sciences and BIO5 Institute, University of Arizona, Tucson, Arizona, United States of America; 3 National Key Laboratory of Plant Molecular Genetics, CAS Center for Excellence in Molecular Plant Sciences, Institute of Plant Physiology and Ecology, Chinese Academy of Sciences, Shanghai, China; 4 International Institute of Tropical Agriculture (IITA), Nairobi, Kenya; 5 Genus plc, DeForest, Wisconsin, United States of America; Pohang University of Science and Technology, REPUBLIC OF KOREA

## Abstract

Cassava (*Manihot esculenta*) is an important tropical subsistence crop that is severely affected by cassava brown streak disease (CBSD) in East Africa. The disease is caused by *Cassava brown streak virus* (CBSV) and *Ugandan cassava brown streak virus* (UCBSV). Both have a (+)-sense single-stranded RNA genome with a 5’ covalently-linked viral protein, which functionally resembles the cap structure of mRNA, binds to eukaryotic translation initiation factor 4E (eIF4E) or its analogues, and then enable the translation of viral genomic RNA in host cells. To characterize cassava eIF4Es and their potential role in CBSD tolerance and susceptibility, we cloned five eIF4E transcripts from cassava (accession TMS60444). Sequence analysis indicated that the cassava eIF4E family of proteins consisted of one eIF4E, two eIF(iso)4E, and two divergent copies of novel cap-binding proteins (nCBPs). Our data demonstrated experimentally the coding of these five genes as annotated in the published cassava genome and provided additional evidence for refined annotations. Illumina resequencing data of the five eIF4E genes were analyzed from 14 cassava lines tolerant or susceptible to CBSD. Abundant single nucleotide polymorphisms (SNP) and biallelic variations were observed in the eIF4E genes; however, most of the SNPs were located in the introns and non-coding regions of the exons. Association studies of non-synonymous SNPs revealed no significant association between any SNP of the five eIF4E genes and the tolerance or susceptibility to CBSD. However, two SNPs in two genes were weakly associated with the CBSD responses but had no direct causal-effect relationship. SNPs in an intergenic region upstream of *eIF4E_me* showed a surprising strong association with CBSD responses. Digital expression profile analysis showed differential expression of different eIF4E genes but no significant difference in gene expression was found between susceptible and tolerant cassava accessions despite the association of the intergenic SNPs with CBSD responses.

## Introduction

The eIF4E family of eukaryotic initiation factor proteins play a crucial role in the initiation of cap-dependent translation of any RNA messenger. eIF4E or its homologue binds to the 7-methylguanosine (m^7^G) cap at the 5’ end of the mRNA in eukaryotes [1,2]. It also binds to eIF4G and eIF4A to form the eIF4F complex, which together with eIF4B and the poly(A)-binding protein form an active mRNA recruiting complex and deliver an mRNA to the 43S pre-initiation complex via protein-protein interaction between eIF4G and eIF3 of the 43S complex [2]. There are at least two distinct and redundant isoforms of eIF4F in higher plants, eIF4F containing eIF4E and eIF4G, and eIF(iso)4F containing eIF(iso)4E and eIF(iso)4G [3]. Although they are considered equivalent in *in vitro* assays, the two isomers have different *in vivo* specificity for certain classes of capped mRNAs [4,5]. In eukaryotes, especially in dicots, several genes code for a small family of eIF4E and eIF(iso)4E proteins [1]. These homologues presumably provide both redundancy and differential regulation during mRNA translation. eIF4E is more elastic than other translation initiation factors. Knockout and/or down-regulation of eIF4E or eIF(iso)4E in *Arabidopsis* is tolerated and plants show little sign of impairment, however depletion of both leads to a dwarf phenotype [6,7]. In addition to these two types of canonical translation initiation factors, plants also encode novel cap binding proteins (nCBP) [1,8,9] that interact with eIF4G and actively participate in mRNA translation [9].

eIF4E and its homologues are not only required for the translation of capped mRNA but also for the translation of (+)-stranded viral RNA genomes with a proteinaceous cap analog: a 5’-covalently-linked viral protein (VPg). Instead of a normal m^7^G cap, many (+)-sense, single-stranded RNA viruses possess a VPg at the 5’ terminus of the viral genomic RNA. These viruses include plant-infecting *Potyviridae* and *Seconviridae* families and *Sobemovirus* genus [10], and vertebrate-infecting *Picornaviridae* and *Caliciviridae* families [11], many of which are serious pathogens of plants, animals, and humans. With the exception of *Picornaviridae*, translation of the viral genomic RNA in these viruses depends on the VPg and its specific interaction with eIF4Es [10,11].

Translation of viral genomic RNA of (+)-sense, single-stranded RNA virus is the most critical first step upon infection. The obligate dependence of these viruses on the translation initiation factors has been demonstrated through the identification and characterization of a number of natural recessive resistance (R)-genes against plant viral pathogens [12–14]. Among the 14 virus-resistance genes cloned and characterized so far, eight of them are mutant alleles of eIF4E and two of them are mutant alleles of eIF4G most of which are mutant alleles of eIF4E and eIF4G [15]. Partial redundancy of eIF4E and eIF4G makes it possible for plants to exhibit recessive resistance to viruses while not affecting the health of the host plants [13,16].

*Cassava brown streak virus* (CBSV) and *Ugandan cassava brown streak virus* (UCBSV) are members of *Potyviridae* carrying a genome-linked VPg [17–19]. Both viruses can singularly or together infect cassava (*Manihot esculenta* Crantz) and cause cassava brown streak disease (CBSD). Cassava is a major staple food crop for 800 million people in Africa, South America, and Southeast Asia. CBSD can cause yield losses of up to 100% and total economic losses of more than $100 million each year [20]. The disease is thus considered as a major threat to sustainable production of cassava and as one of the seven most serious threats to global food security since its reemergence in 2000 [20,21].

There is no known natural immunity to CBSD in cultivated cassava, but some landraces or accessions are tolerant to CBSD [22]. As the VPgs of both CBSV and UCBSV are expected to interact with eIF4E or its homologues during infection, it is conceivable that natural variations in cassava eIF4E may contribute to the reported CBSD tolerance. In order to understand the diversity of the cassava eIF4E proteins and their association with tolerance and susceptibility to CBSD, we cloned and sequenced all members of cassava eIF4E family of proteins and analyzed genetic variations in selected accessions with tolerant or susceptible responses to CBSD. Cassava possessed a single *eIF4E* gene, two highly conserved *eIF(iso)4E* genes, and two divergent nCBP genes. Among the five genes, *eIF4E* and one copy of each *eIF(iso)4E* and nCBP genes were expressed at a significantly higher level than the remaining two genes. Examination of single nucleotide polymorphisms (SNPs) revealed biallelic variations among the resequenced genomes of 14 representative lines of cassava accessions indicative the heterozygous nature of this outcrossing, clonally propagated crop. Co-existence of both alleles of the same eIF4E inherited from both parents was apparent in some landraces. There were abundant SNPs in the eIF4E genes, but most SNPs were located in the non-coding regions. There was no significant association between non-synonymous SNPs in eIF4E genes and the tolerance or susceptibility to CBSD in the surveyed genotypes, however a stronger association was observed between SNPs in a region upstream of the *eIF4E* gene and CBSD responses.

## Materials and methods

### Cassava materials and RNA isolation

Cassava accession TMS60444 was grown for three months under long-day condition (16/8 h light/dark cycle, 28°C, 150 μmol/m^2^s light intensity) in a greenhouse. Expanded leaves of ~4-5cm in length were harvested, snap-frozen in liquid nitrogen, and stored at -80°C prior to RNA extraction. Total RNA was extracted from 100 mg of leaf tissue using PureLink^®^ Plant RNA Reagent following the protocol provided by the manufacturer (Ambion, Waltham, USA). The RNA preparation was subsequently treated with DNase I to remove any potential contaminating genomic DNA, followed by phenol-chloroform extraction and ethanol precipitation. The RNA quality was evaluated by agarose gel fractionation and RNA concentration was measured with NanoDrop^®^ ND-1000 (NanoDrop Technologies, Wilmington, USA).

### Reverse transcription-PCR amplification of eIF4E mRNA transcripts

First-strand cDNA synthesis using SuperScript III Reverse transcriptase (Invitrogen, Waltham, USA) was carried out essentially as described by the manufacturer. The reaction contained 1 μg total RNA and 250 pmol oligo BamHI(dT)_30_ primer (Table 1) in a 10 μl volume. Reverse transcription was carried out at 42°C for 5 minutes followed by 50°C for 40 minutes, and terminated by incubation at 70°C for 10 minutes. Sequences corresponding to the open reading frames (ORFs) of eIF4E genes were amplified by PCR using gene-specific forward and reverse primers (Table 1), and mRNA sequences containing the complete 3’ untranslated region (UTR) of genes were also amplified with the specific upstream primers and a downstream BamHI(dT)_30_ primer (Table 1). PCR was performed in a 20 μl reaction volume containing 10 unit Phusion DNA polymerase (NEB, Ipswich, MA), 1 μl of 1:5 diluted cDNA template, 1X Phusion PCR buffer, 5 μM each of upstream and downstream primers, and 250 nM dNTP with the following cycling condition: 98°C for 1 minute; 35 cycles of 98°C for 15 seconds, 56°C for 15 seconds, and 72°C for 45 seconds; and finally 72°C for 5 minutes. Primers were designed according to five annotated eIF4E transcripts identified in the draft cassava genomic sequence (*Manihot esculenta* v4.1) published in Phytozome (http://phytozome.jgi.doe.gov) in 2013, prior to the availability of the current cassava genome V6.1 (Table 1). The forward primers consisted of two protective bases and *Eco*RI restriction site (bolded in the table) in addition to specific nucleotides corresponding to sequences starting at ATG codon (italicized in the table) of each eIF4E candidate gene. The reverse primers consisted of three protective bases and *Bam*HI restriction site (bolded in the table) in addition to specific nucleotides complementary to sequences beginning with a termination codon (italicized in the table) of each eIF4E candidate genes. The optimal Tm_50_ for each primer was targeted at 56°C to 58°C. Two transcripts annotated in cassava genome V4.1, cassava4.1_013223m and cassava4.1_013732m, were found to encode 38 and 50 more amino acids than their cognate homologues in other plants, and the downstream AUG starting codons of these two genes were found in a better translation initiation context than the predicted AUG codons. Therefore, two additional primers were designed to amplify the revised, shorter CDS for the two genes (Table 1, 013223lF, 013223sF, 013732lF, and 013732sF).

**Table 1:** Primers used to amplify coding sequences of cassava eIF4E and its homologues.

Primers	Sequences	Targeted eIF4E locus
**013223lF**	5’ CG**GAATTC***ATG*GATACTCTTGGTCAAATTAAT 3’	Manes.17G063100 (cassava4.1_013223)
**013223sF**	5’ CG**GAATTC***ATG*GCGGCGGAAGAGCCATTGAAA 3’	
**013223R**	5’ GCC**GGATCC***CTA*GTAATAGGCAGGGCTCATGGA 3’	
**013732lF**	5’ CG**GAATTC***ATG*AATAAGAGCGCTAGAGGTGAA 3’	Manes.09G140300 (cassava4.1_013732)
**013732sF**	5’ CG**GAATTC***ATG*GAGATCACAGAGGAGGAAACA 3’	
**013732R**	5’ GCC**GGATCC***TTA*ACCTCTCAACCAAGTGTTTCT 3’	
**015501F**	5’ CG**GAATTC***ATG*GAGATCACTGAGAAGAAGGAT 3’	Manes.08G145200 (cassava4.1_015501)
**015501R**	5’ GCC**GGATCC***TTA*TCCTCTCAACCATGTGTTTCT 3’	
**016601F**	5’ CG**GAATTC***ATG*GCAACCGAAACAGCAACAGAA 3’	Manes.03G160000 (cassava4.1_016601)
**016601R**	5’ GCC**GGATCC***TTA*TACATTGTATCGGCCTTTGAC 3’	
**016620F**	5’ CG**GAATTC***ATG*GCAAGCGAAACCGCAATAGAA 3’	Manes.15G044900 (cassava4.1_016620)
**016620R**	5’ GCC**GGATCC***TTA*TACATTATATCGGCCTTTGGC 3’	
**BamHIdT**	5'–GCTAGGATCCTTTTTTTTTTTTTTTTTTTTTTTTTTTTTT-3’	Poly(A) tail

### Cloning and sequencing analysis of cloned eIF4E transcripts

The PCR-amplified cDNA fragments were first digested with *Eco*RI and *Bam*HI and cloned into the plasmid pGADT7 (Clontech, Mountain View, CA) previously digested with the same two enzymes. Clones with predicted sizes of inserts were verified by colony PCR with the Matchmaker 5' AD LD-Insert Screening Amplimer primer and Matchmaker 3' AD LD-Insert Screening Amplimer (Clontech, Mountain View, CA) as previously described [23]. Three to five independent clones from each candidate eIF4e gene were sequenced in both directions using Applied Biosystems 3730 DNA Analyzers at the University of Arizona Genetics Core Facility. Sequencing reads were assembled using CodonCode Aligner V4.2 (CodonCode, Centerville, MA).

### Phylogenetic analysis

Predicted protein sequences of the cassava eIF4E genes were analyzed together with representative eIF4E proteins retrieved from NCBI Genbank. Sequences were aligned using the default parameters of the ClustalX 2.1 program [24]. Gaps are manually inspected and adjusted if necessary. A model test was performed to identify most optimized parameters for phylogenetic analysis. Maximum likelihood inference of phylogenetic relationships was carried out using the Jones-Taylor-Thornton model with gamma-distributed rate variation (JTT+G) as implemented in MEGA version 7.0 [25]. Phylogenetic trees were then visualized using the same program.

### Analysis of resequenced cassava landraces/accession

Nucleotide variations in the Illumina resequenced whole genomes of selected cassava accessions were visualized and examined using the Integrated Genome Viewer Version 2.3 [26]. SNPs in the five eIF4E genes and the surrounding regions (+/- 5kb) were retrieved from the Genome Diversity V12 of the Biomart in Phytozome (http://phytozome.jgi.doe.gov). Association studies were conducted in Plink V1.9 [27]. Odd ratios of disease responses as a linear function of the underlying nucleotide variations was estimated with logistic regression models, and the probability of each SNP associated with the CBSD tolerance and susceptibility responses was assessed with the Chi-square test and validated by 100,000 Monte Carlo permutations. The significance threshold for the association test was corrected with the Bonferroni method, taking into consideration of the five genes (linkage groups) and two phenotypes being tested. The adjusted *p* value threshold for a significant SNP is set at 0.005 (α = 0.05/(5 linkage groups x 2 phenotypes)) regardless the number of SNPs in the five genes.

### Digital expression profile analysis of eIF4E gene expressions

Illumina RNAseq data generated from CBSD-susceptible Albert and CBSD-tolerant Kaleso cassava line were retrieved from the NCBI SRA depository (accessions SRR1213744- SRR1213747) [28]. 454 Life Sciences transcriptomic sequencing data of eight CBSD-susceptible and CBSD–tolerant cassava accessions were also retried from the NCBI SRA depository (accessions SRR955444-SRR955447, SRR955449, SRR955450, SRR955453, and SRR955456) [29]. Adapter sequences were removed and low quality bases and reads were clipped and removed before sequencing reads were mapped against the cassava genome using Bowtie version 2.2 [30]. Cuffdiff program in the Cufflnks suite [31] was then used to analyze differential gene expressions of the Illumina data set. Long reads of the 454 sequencing data were counted directly and normalized to total mapped reads for statistical analysis of gene expressions in MATLAB version R2017a. All the bioinformatics analyses were conducted in the Discovery Environment of Cyverse (Cyverse.org).

## Results

### Analysis of five cassava eIF4E transcripts

**Fig 1:**
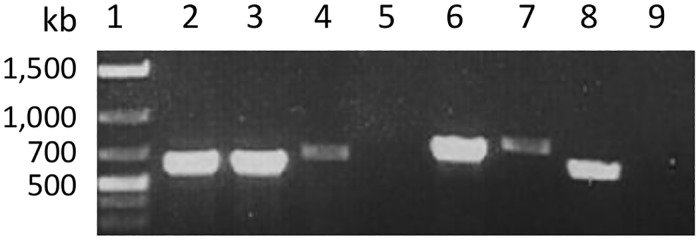
RT-PCR amplification of five cassava eIF4E ORFs with gene-specific primers. Total RNA was extracted from TMS60444 cassava line. The first-strand cDNA was synthesized using SuperScript^™^ III Reverse Transcriptase (Invitrogen) and PCR was performed using Phusion High-Fidelity DNA polymerase (New England Biolabs) and primers indicated in Table 1. Lane 1: DNA marker; Lane 2: 016601 CDS amplified with primers 016601F and 016601R; Lane 3: 016620 CDS amplified with primers 016620F and 016620R; Lane 4: 015501 CDS amplified with 015501F and 015501R, Lane 5: 013223 CDS amplified with primers 013223lF and 013223R, Lane 6, 013223 CDS amplified with primers 013223sF and 013223R; Lane 7: 013732 CDS amplified with 013732lF and 013732R; Lane 8: 013732 CDS amplified with 013732sF and 013732R; and Lane 9: negative water control.

The cassava genome assembly V6.1 (phytozome.jgi.doe.gov) [32,33] annotated five genes with six predicted transcripts encoding for proteins with domains conserved in the eIF4E superfamily [34]. These are Manes.03G160000.1, Manes.08G145200.1, Manes.09G140300.1, Manes.15G044900.1, Manes.17G063100.1, and Manes.17G063100.2. cDNA was synthesized with a oligo(dT) primer containing a BamHI restriction site at the 5’ end. DNA fragments corresponding to the predicted sizes were amplified with either the gene specific primers or gene-specific forward primers and oligo(dT) reverse primer (Table 1) for the all but one primer pair (Fig 1). Upon further analysis, primer 013223lF was located outside of the annotated transcripts Manes.17G063100.1 and Manes.17G063100.2 in cassava genome V6.1, and therefore 013223lF and 013223R failed to amplify a correct cDNA fragments.

Nine cDNA haplotype sequences of eIF4E mRNA transcripts were assembled from 30 independent clones sequenced and submitted to the NCBI Genbank (Accessions KY673619-KY673627). The transcripts were exactly as predicted by the annotated Cassava Genome V6.1 [32] at the following loci: Manes.03G160000, Manes.08G145200, and Manes.17G063100. Two alleles of *eIF(iso)4E_me1* (accessions KY673619 and KY673620) were obtained from the Manes.03G160000 locus, and they differed by four SNPs (C/T at nt 144, G/C at nt 146, C/T at nt 180, and A/T at nt 466). The substitution at nucleotides 146 and 466 also resulted in T49S and S156T changes in the predicted protein sequences, respectively. Two alleles of *nCBP_me2* (KY673623 and KY673624) were sequenced from the Manes.08G145200 locus. They differed by two nucleotides (A/G at nt 450 and C/T at nt 520) but encoded identical protein sequences. A single allele of *eIF4E_me1* mRNA sequence (KY673627) identical to the annotated transcript Manes.17G063100.1 was obtained but no copies of the predicted alternate transcript, Manes.17G063100.2, was found in eight cDNA clones screened.

Analysis of the transcripts from two remaining eIF4E loci also confirmed annotations in cassava genome V6.1 but with some discrepancies. Two transcripts of *eIF(iso)4E_me2* with different 3’ untranslated regions (UTRs) were sequenced from the Manes.15G044900 locus (accessions KY673621 and KY673622). KY673621 has a 3’ UTR 26 nucleotides shorter than that of KY673622, and both transcripts were shorter by 125 and 99 nucleotides in the 3’ UTR than the predicted transcript Manes.15G044900.1. Both KY673621 and KY673622 were likely products of real transcription termination and polyadenylation events as the terminal A nucleotide in TTGATTTTCCGCA of KY673621 was predicted by PASPA [35] as an authentic polyadenylation site. A similar motif of TTAATTTTGGATCA was also found immediately upstream of the polyadenylation site in KY673622. Two SNPs, C/T at nt 177 in the ORF and C/T at nt 814 in the 3’ UTR, were also present in the *eIF(iso)4E_me2* transcripts. Two sequences of *nCBP_me1a* were also cloned using two different 5’ primers: KY673625 contained a 5’ UTR of 151 nucleotides while KY673626 contained the ORF only. Nucleotide 131 (T) of the 5’ UTR in KY673625 is missing from the annotated cassava genome V6.1. KY673625 was 26 nucleotide longer than the predicted transcript (Manes.09G140300.1) and it was apparent that transcription from the locus in TMS60444 started further upstream as the 5’ primer, 013732lF, upstream of the predicted transcribed region was able to amplify cDNA efficiently (Fig 1, lane 7).

Based on the sequencing analysis, we concluded experimentally that there were five members of the eIF4E family genes encoded in the TMS60444 cassava genome. The mRNA transcribed from loci Manes.03G160000, Manes.08G145200, Manes.09G140300, Manes.15G044900, Manes.17G063100 encoded for proteins of 200, 228, 223, 233, 200 amino acids, respectively.

### Cassava encodes three families of eIF4E proteins

**Fig 2:**
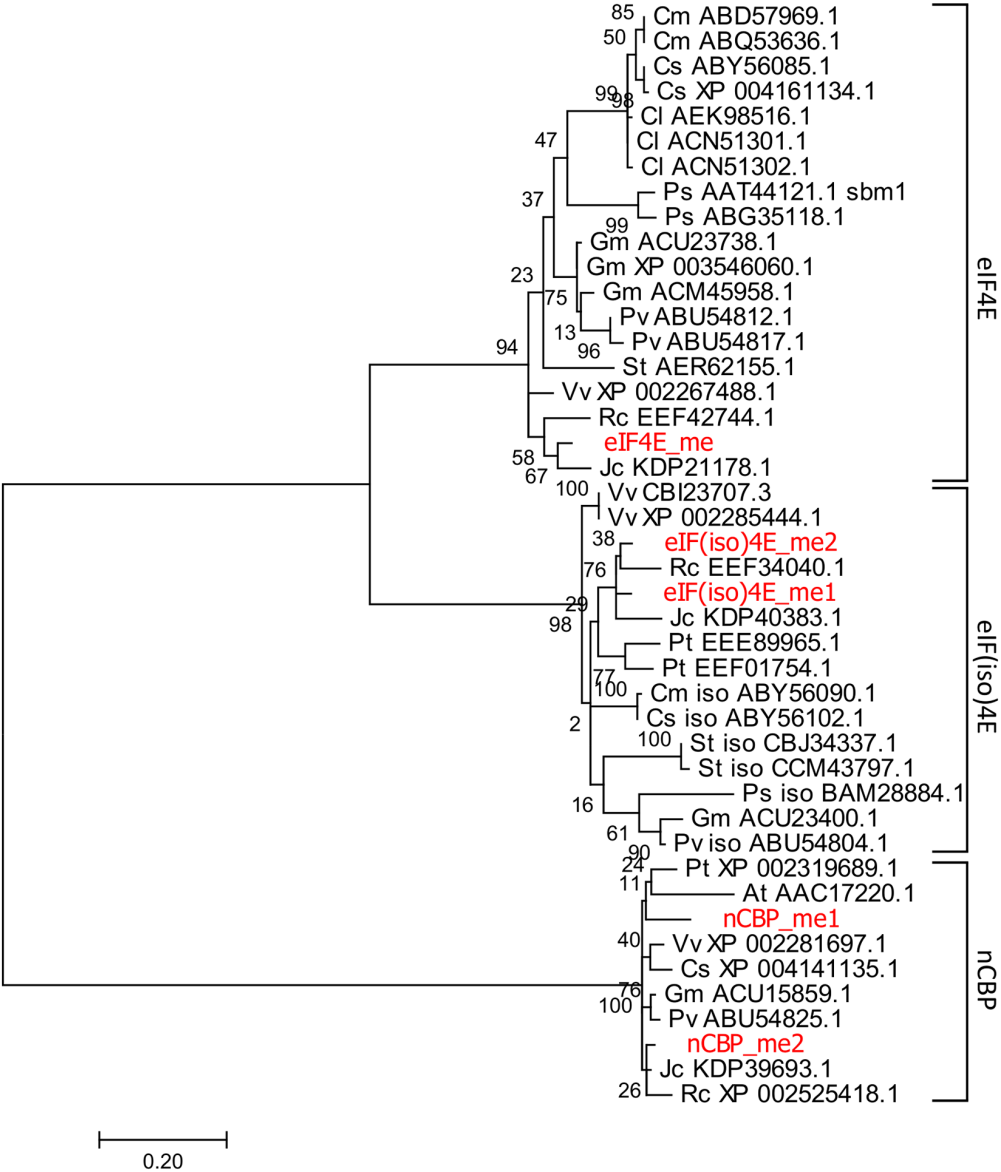
Phylogenetic analysis of eIF4E superfamily proteins. The phylogenetic tree was inferred by using the Maximum Likelihood method based on the Jones-Taylor-Thornton matrix-based model with gamma-distributed rate variation (JTT+G) as implemented in MEGA version 7.0 [25]. A discrete Gamma distribution was used to model evolutionary rate differences among sites (6 categories, +*G*, parameter = 1.7002). The tree with the highest log likelihood (-2897.9607) is shown. The tree is drawn to scale, and the scale bar represents 0.2 substitution per site. The percentage of replicate trees in which the associated taxa clustered together in the bootstrap test (1000 replicates) are shown next to the branches. The analysis involved 42 protein sequences. All positions containing gaps and missing data were eliminated. Protein sequences of additional eIF4E family proteins were retrieved from NCBI. Proteins are identified with two letters representing the initials of genus and species, followed by NCBI accession number. At, *Arabidopsis thaliana*; Ca: *Capsicum annuum*; Cl, *Citrullus lanatus*; Cm, *Cucumis melo*; Cs, *Cucumis sativus*; Gm, *Glycine max*; Jc, *Jatropha curcas*; Ps, *Pisum sativum*; Pt, *Populus trichocarpal*; Pv, *Phaseolus vulgaris*; Rc, *Ricinus communis*; St, *Solanum tuberosum*; and Vv, *Vitis vinifera*. Clustering of three families of eIF4E proteins are indicated and labeled: eIF4E, eIF(iso)4E, and novel cap binding protein (nCBP).

Phylogenetic analysis clearly showed the presence of three distinct families of eIF4E proteins encoded by the cassava genome (Fig 2). There were two eIF4(iso)4E proteins (eIF4(iso)4E_me1 and eIF4(iso)4E_me) and two novel cap binding proteins (nCBP_me1 and nCBP_me2), but only a single eIF4E_me protein was present. The single cassava eIF4E_me protein was most closely related to the eIF4E protein from *Jatropha curcas* (86% amino acid identity) of the *Euphorbiaceae*, the same family that cassava belongs to. The eIF4E proteins of *Euphorbiaceae* and *Fabaceae* form a distinct clade in the phylogenetic analysis (Fig 2). The two cassava eIF(iso)4E proteins were closely related to each other, sharing 93% amino acid identity and 97% amino acid similarity. A recent duplication event followed by divergence may account for this high degree of similarity. They are most closely related to eIF(iso)4E proteins from *J*. *curcas* (88% identity) and *Ricinus communis* (87~88% identity) of the *Euphorbiaceae* family, and are co-clustered in the phylogenetic tree. The two nCBPs of cassava were more divergent from each other, with 88.1% amino acid identity and 90.1% amino acid similarity. These two cassava nCBPs segregated with other nCBPs to form a large clade but there is no apparent subclustering within families of plant species. The *Arabidopsis* nCPB (At_AAC17220), which was the first nCBP (4EHP in human) identified and characterized [9], also segregated into the same large clade of nCBP. The large divergence of the two nCBPs perhaps reflected an earlier duplication event and subsequent functional diversification over time in plants.

All members of the cassava eIF4E proteins possessed two highly conserved aromatic amino acid residues involved in the cap-binding via base stacking and hydrogen bonding [36]. An exception was observed in nCBP. Both the eIF4E_me and eIF(iso)4E_me1 and eIF(iso)4E_me2 contained the tryptophan residues at positions equivalent to the Trp^56^ and Trp^102^ of human eIF4E while nCBPs (nCBP_me1 and nCBP_me1) substituted the tryptophan residue with an aromatic tyrosine at the Trp^56^ position (Figure B in S1 File). Since the *Arabidopsis thaliana* nCBP possessed tyrosine at the same position and have been demonstrated to have cap-binding activities [36], the aromatic tyrosine substitution at the Trp^56^ is assumed not to affect the cap-binding of these proteins. All original cap-binding proteins had eight conserved tryptophans at relative positions [37,38]. While all the eight tryptophans were present in the cassava eIF4E and eIF(iso)4E proteins, the first and third typtophans in nCBP_me2 were replaced with a phenylalanine and a tyrosine, respectively, and the first three tryptophans in nCBP_me1 were replaced with a phenylalanine, a leucine, and a tyrosine, respectively (Figure B in S1 File). The changes in the conserved tryptophan residues suggested a significant deviation of the roles of nCBPs, especially of nCBP_me1.

### Sequence variations in eIF4E genes across different cassava varieties

Compatible interaction between eIF4E proteins of host and potyviral VPg proteins is essential for the translation of the positive-sense genomic RNA and initiation of a successful infection. This is demonstrated by a large number of natural mutant alleles of eIF4E and eIF4G genes that provide recessive resistance to viruses with VPgs covalently linked to their genomes [13,16,39–41]. The causal viruses of CBSD, CBSV and UCBSV, are potyviruses having a genome covalently linked to a VPg. There is no known immunity in domesticated cassava lines, but some show a tolerant phenotype to CBSD. Because of the importance of eIF4E in potyviral infection, there is a possibility that genetic variations in the eIF4E gene may be associated with tolerance in some cassava lines. To assess this possibility, SNPs were identified in cassava accessions with known susceptibility or tolerance to CBSV and UCBSV, and an association study was performed to correlate any SNP to the CBSD phenotypes. Fourteen cassava accessions with known phenotypes to CBSV/UCBSV infection were selected for this analysis (Table 2) [22,29,42–44]. In the typical susceptible lines, viruses accumulate rapidly in the infected plants and symptoms become severe, whereas in the tolerant lines, virus load is restricted and symptoms are limited [22]. The phenotypes were observed under field conditions in Tanzania and/or Uganda where one or both cassava brown streak viruses could infect.

**Table 2:** Response of selected cassava accessions to CBSV/UCBSV infection*.

Cultivar	Tolerant/Susceptible
**Albert**	Susceptible
**AM560**	Susceptible
**AR37-80**	Susceptible
**Kibandameno**	Tolerant
**Kiroba**	Tolerant
**MCOL22**	Susceptible
**Mkombozi**	Susceptible
**Muzege**	Tolerant
**Namikonga**	Tolerant
**Nachinyaya**	Tolerant
**TME3**	Susceptible
**TME7**	Susceptible
**TMS30572**	Susceptible
**TMS60444**	Susceptible

* Phenotype data were obtained from [22,29,42–44].

Polymorphic sites in the shot-gun resequenced genomes of 61 cassava accessions [32] were retrieved from Phytozome V12. Overall, there were 861 SNP positions in the five eIF4E genes across 61 cassava accessions, ranging from 110 SNP sites in *eIF4E_me* to 221 SNP sites in *nCBP_me1* (Table A and Figure C in S1 File). However, most of the SNPs were located in the introns of the genes, and only 252 SNPs were located in in the exons (Table A and Figure C in S1 File). The coding sequences (CDSs) of these genes harbored only 102 SNP sites, about 12% of all SNP sites in the genes, while the remaining 150 SNPs were found in the 5’ and 3’ UTRs. Half of the SNP in the CDSs contained synonymous nucleotide substitutions while the other half contained non-synonymous nucleotide changes (Table A in S1 File). The SNP sites were unevenly distributed across five cassava eIF4E genes, the largest number of SNP sites was found in eIF4E_me (30) while the least was found in eIF(iso)4E_me2 (13). The uneven distribution of SNPs across and among these genes, and the heterozygous nature of cassava accessions was clearly evident when the genetic variations in the 5 eIF4E genes in representative genomes were visualized with the Integrative Genomics Viewer version 2.3 [26] (Figure A in S1 File). This analysis also showed the homozygous and heterozygous status of various eIF4E genes across different cassava genotypes. While some eIF4E genes are homozygous in some varieties, other eIF4E genes are heterozygous in other varieties. Accession AM560, a partial inbred line [33], is the only exception with all five eIF4E genes being homozygous.

### Two SNPs in two eIF4E genes are weakly associated with CBSD responses

eIF4E and VPg interaction occurs at the protein-protein level. Therefore, we initially focused on the analysis of 51 non-synonymous SNP sites resulting in a change in the eIF4E proteins. These SNPs were extracted from 14 cassava accessions that have known tolerance and susceptible responses upon exposure to CBSV/UCBSV (Table 2) [22,29,42–44]. The genome diversity dataset of *M*. *esculenta* V6.1 in Phytozome V12 included cultivated cassava as well as five accessions of *M*. *glaziovii* and *M*. *pseudoglaziovii* [32,45], the wild relatives of *M*. *esculenta*. Due to the large genetic distance, a majority of SNP sites were contributed by these wild cassava relatives. Only 13 out of 51 non-synonymous SNP sites were informative in the subset of data of the 14 cassava accessions after missing data and monomophic sites were excluded (Table 3, Table B in S1 File). The five cassava eIF4E genes contained 0, 1, 1, 3, and 8 informative SNPs for nCBP_me2, eIF(iso)4E_me2, nCBP_me1, eIF(iso)4E_me1, and eIF4E_me, respectively (Table 3). When these SNPs and the disease responses in these cassava accessions were modeled with unconditional logistic regression for their association odd ratios, none of the SNPs was statistically associated with the disease phenotypes at the Bonferroni corrected *p* value of 0.005. However, two of the SNPs were found to be weakly related to CBSD disease responses: one at p value < 0.05, and one at *p* value < 0.10 (Table 4). The non-synonymous SNP in the *nCBP_me1* gene, a C to T transition at Chr09_25948588, manifested a K40E change in the protein. The other non-synonymous SNP, A to T transversion at Chr17_20187344, resulted in amino acid changes of L223H in *eIF4E_me*, respectively. It was difficult to interpret the significance of these associations due to the high p values. The adjusted threshold *p* value of SNP associations depended on the number of SNPs and/or linkage regroups and the number of phenotypes being test (the number of multiple tests) with the Bonferroni correction method. In this analysis, we adjust the significant threshold value at 0.005, which was derived from the standard α = 0.05, divided by the five linkage groups (genes) and the two phenotypes (tolerance and susceptibility). In a normal genome wide association study with 500,000 to 1 million SNPs, the adjusted threshold *p* value is set at 10^−7^ to 10^−8^ for an SNP to be considered significant.

**Table 3:** Distribution and characteristics of SNP sites in five eIF4E genes and neighboring regions in 14 cassava accessions*.

Regions	eIF(iso)4E_me1	nCBP_me2	nCBP_me1	eIF(iso)4E_me2	eIF4E_me	Total SNP
**Downstream**	89	69	57	152	187	554
**Intron**	21	16	30	71	36	174
**Non-synonymous**	3	0	1	1	8	13
**Synonymous**	2	1	0	4	4	11
**Upstream**	82	54	56	181	166	539
**3' UTR**	13	3	9	9	3	37
**5' UTR**	0	0	2	0	1	3
**Total**	210	143	155	418	405	1331

* Locus information for the eIF4E genes in the V6.1 of the cassava genome are as follows: eIF(iso)4E_me1: 03G160000, nCBP_me2: 08G145200, nCBP_me1: 09G140300, eIF(iso)4E_me2: 15G044900, eIF4E_me: 17G063100. SNPs were extracted from the 16 cassava accessions listed in Table 2.

**Table 4:** Association of eIF4E non-synonymous SNPs with CBSD-susceptible and tolerant phenotypes.

Genes	Chromosome	Location	SNP^1^	aa Changes	*P* value	AIC	BIC
**eIF(iso)4E_016601m**	Chr03	25396170	G/G(14), G/C(1),./.(1)	K26N	0.420	20.8	22.2
		25396238	G/G(12), G/C(3),./.(1)	S49T	0.150	19.3	20.7
		25396987	A/A(11), A/T(2),./.(3)	T156S	0.200	18.4	19.6
**nCBP_013732m**	Chr09	25948588	C/C(6), C/T(8), T/T(2)	K40E	0.097^2^	21.1	22.7
**eIF(iso)4E_016620m**	Chr15	3318172	C/C(6), C/T(7), T/T(1),./.(2)	G165S	0.160	18.8	20.1
**eIF4E_013223m**	Chr17	20187344	A/A(12), A/T(3), T/T(1)	L223H	0.033^3^	19.3	20.9
		20187753	T/T(15), T/C(1)	N174S	0.120	21.4	22.9
		20188018	A/A(14), A/C(1),./.(1)	V111G	0.091	18.5	20.0
		20188026	C/C(14), C/G(1),./.(1)	K108N	0.091	18.5	20.0
		20189389	C/C(14), C/T(1),./.(1)	A78T	0.130	20.8	22.2
		20189407	T/T(14), T/A(1),./.(1)	T72S	0.130	20.8	22.2
		20189457	T/T(12), T/C(1),./.(3)	Y55C	0.150	19.3	20.4
		20189544	T/T(8), T/A(6), A/A(1),./.(1)	Q26L	0.360	22.2	23.7

^1^Occurrences of the SNP genotypes are denoted in parenthesis../. indicates missing data. AIC and BIC values are used to select the best model to describe the association between the SNP and the phenotypes.

^2^*P*-value is estimated with an overdominant model, which produced the lowest AIC, and BIC values.

^3^*P*-value is estimated with a dominant model, which produced the lowest AIC and BIC values.

### SNPs upstream of *eIF4E_me* are associated with CBSD responses

**Fig 3:**
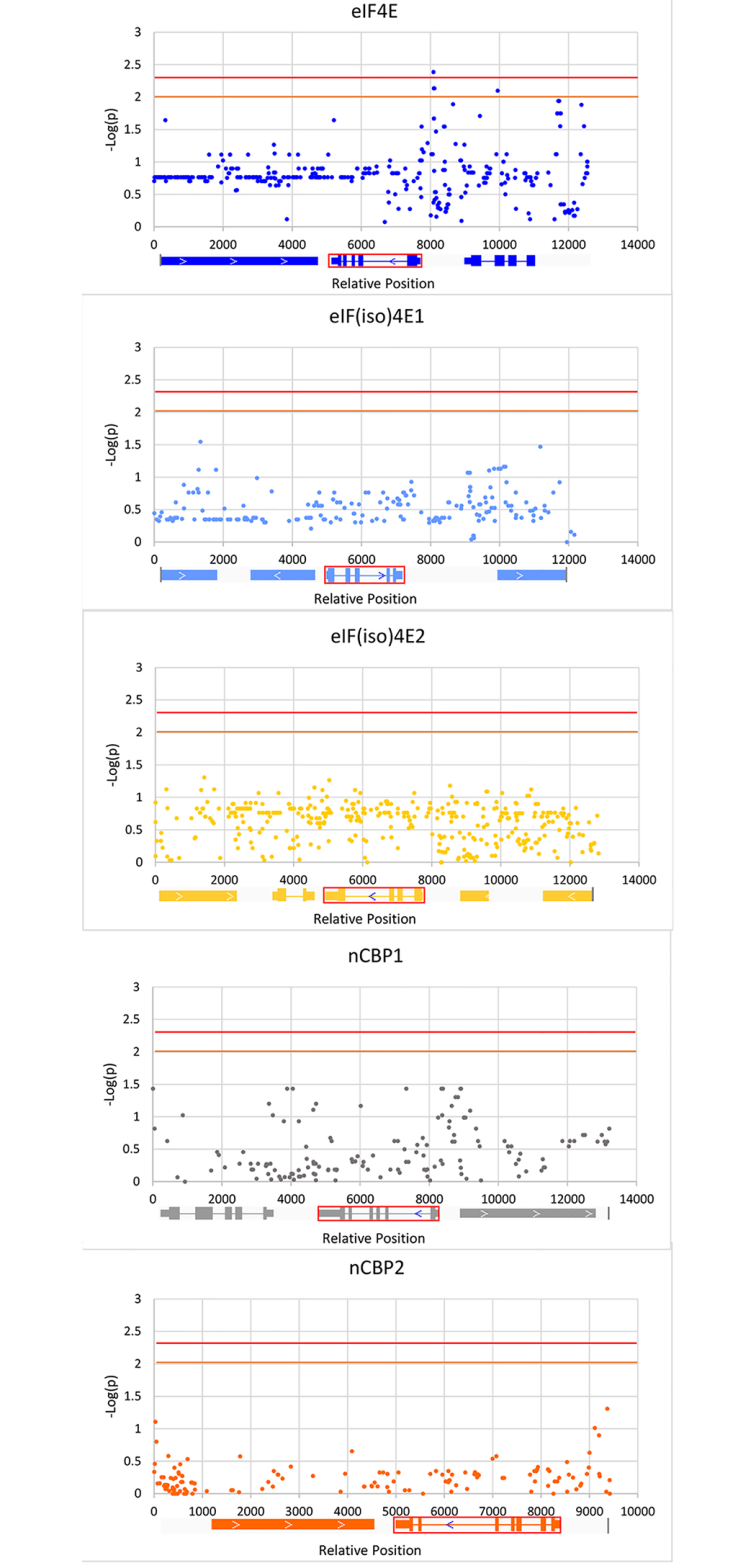
Manhattan scatter plots of probabilities of 1358 SNPs in eIF4E genes and surrounding regions (+/- 5 kb) associated with CBSV responses. Informative SNPs within +/- 5 kb regions of each eIF4E genes were tested for their likelihood to be associated with the susceptible or resistant responses to CBSD. Negative log-transformed probability scores are plotted again the relative distances of each SNP in each chromosomes for each of the SNPs. Horizontal axis represents relative distances of each SNP from each other. SNPs from each eIF4E gene are color-coded. The orange and red lines indicate p-value thresholds of 0.01 and 0.005, respectively. Schematic of the gene model in each locus is depicted below the graph and color-coded. eIF4E gene in each locus in indicated by red rectangles. For each eIF4E gene, exons are represented by rectangle boxes while intros are indicated by lines.

We further examined all the SNPs in the five eIF4E genes and the surrounding 10 kb regions (+/- 5 kb of the genes) (Table A in S1 File). Of the 4388 SNPs, only 1331 were informative while the remaining 3057 SNPs were monomorphic among the 14 cassava lines selected for this study (Table 3). The analysis again revealed no strong association between any SNPs in the eIF4E genes and the disease responses. Interestingly, five SNPs in the 5kb region upstream of the *eIF4E_me* gene showed association with *p* values of <0.01 and >0.001, and one SNP in the intergenic region was smaller than the adjusted *p* value threshold set for this study (*p* = 0.005) (Fig 3, Table C in S1 File). Four SNPs were located in the intergenic region between eIF4E_me and Manes.17G063200, and tightly clustered in a short region of 524 and 547 nucleotides upstream of the *eIF4E_me* transcription initiation site. An additional non-synonymous SNP was found in the coding region of Manes.17G063200, 1865 nucleotides further upstream of the four clustered SNPs (Table C in S1 File). The Manes.17G063200 locus is predicted to encode a protein of 355 amino acids containing a N-terminal domain conserved in 2-oxoglutarate/Fe(II)-dependent dioxygenases and a C-terminal domain conserved in the 2-oxoglutarate and Fe(II)-dependent oxygenase superfamily proteins. The best characterize homolog is a senescence regulated protein 1 (SRP1) in Arabidopsis [46], a protein upregulated in senescing and yellowing leave.

### Digital expression profiles of cassava *eIF4E* genes

**Fig 4:**
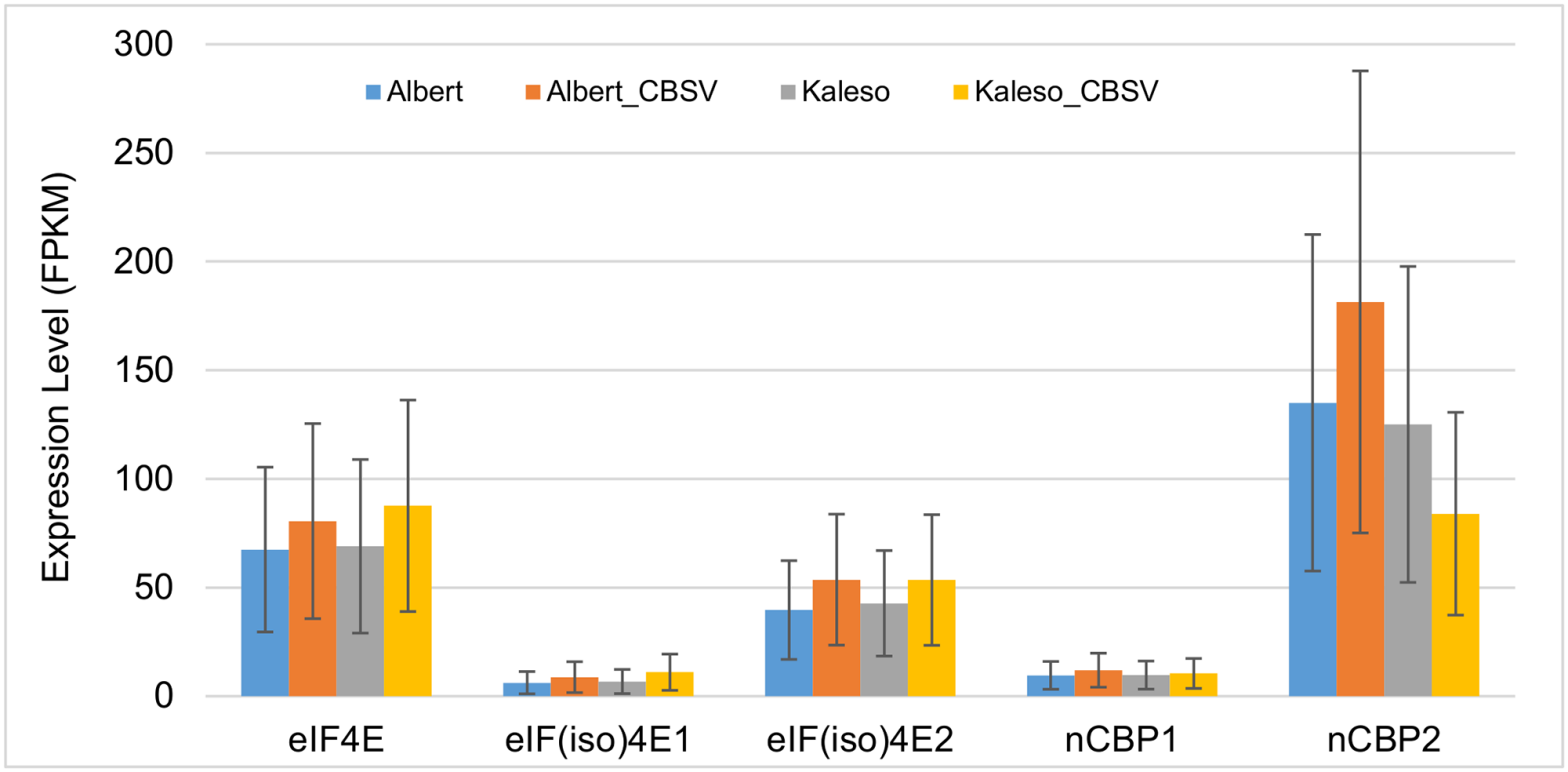
Expression levels of cassava eIF4E genes in the susceptible Albert and the tolerant Kaleso lines. Illimina reads were obtained from the NCBI SRA depository (accessions SRR1213744- SRR1213747) [28]. Cleaned reads were aligned to the cassava genome and differential gene expression were analyzed using the Cuffdiff program [31]. Expression levels of each gene were represented with sequence reads per kilobase per million reads (FPKM). Error bars indicate the 95% confidence interval of the FPKM of each gene. Samples include healthy Albert and Kaleso cassava accessions and CBSV infected Albert (Albert_CBSV) and Kaleso (Kaleso_CBSV).

To ascertain if the marginally associated SNP in the intergenic region of the cassava *eIF4E_me* gene could affect the expression levels of eIF4E in susceptible and resistant cassava accessions, we performed a digital expression profile analysis of all eIF4E genes using high throughput RNAseq data available in the NCBI SRA depository. The first set of data analyzed was obtained from a transcriptomic study of healthy and CBSV-infected Albert and Kaleso cassava accessions [28] (Table D in S1 File). Albert is susceptible to CBSD while Kaleso is tolerant to CBSD. This set of data consisted of transcriptomes from healthy and CBSV-infected cassava accessions and more than 86% of high quality sequence reads were mapped to the cassava genome (Table D in S1 File). Differential gene expression analyses with Cuffdiff [31] showed no significant differences in the expression levels among the five cassava eIF4E genes in the susceptible Albert and the tolerant Kaleso cassava accessions, whether they were healthy or infected with CBSV (Fig 4) (Table E in S1 File). Furthermore, the results revealed that different eIF4E genes were expressed differentially in cassava. *eIF4E*, *eIF(iso)4E2*, and *nCBP2* were expressed at a significantly higher level than *eIF(iso)4E1* and *nCBP1* (Fig 4), suggesting a predominant role of *eIF(iso)4E2* and *nCBP2*, and a complementary role of *eIF(iso)4E1* and *nCBP1*.

**Fig 5:**
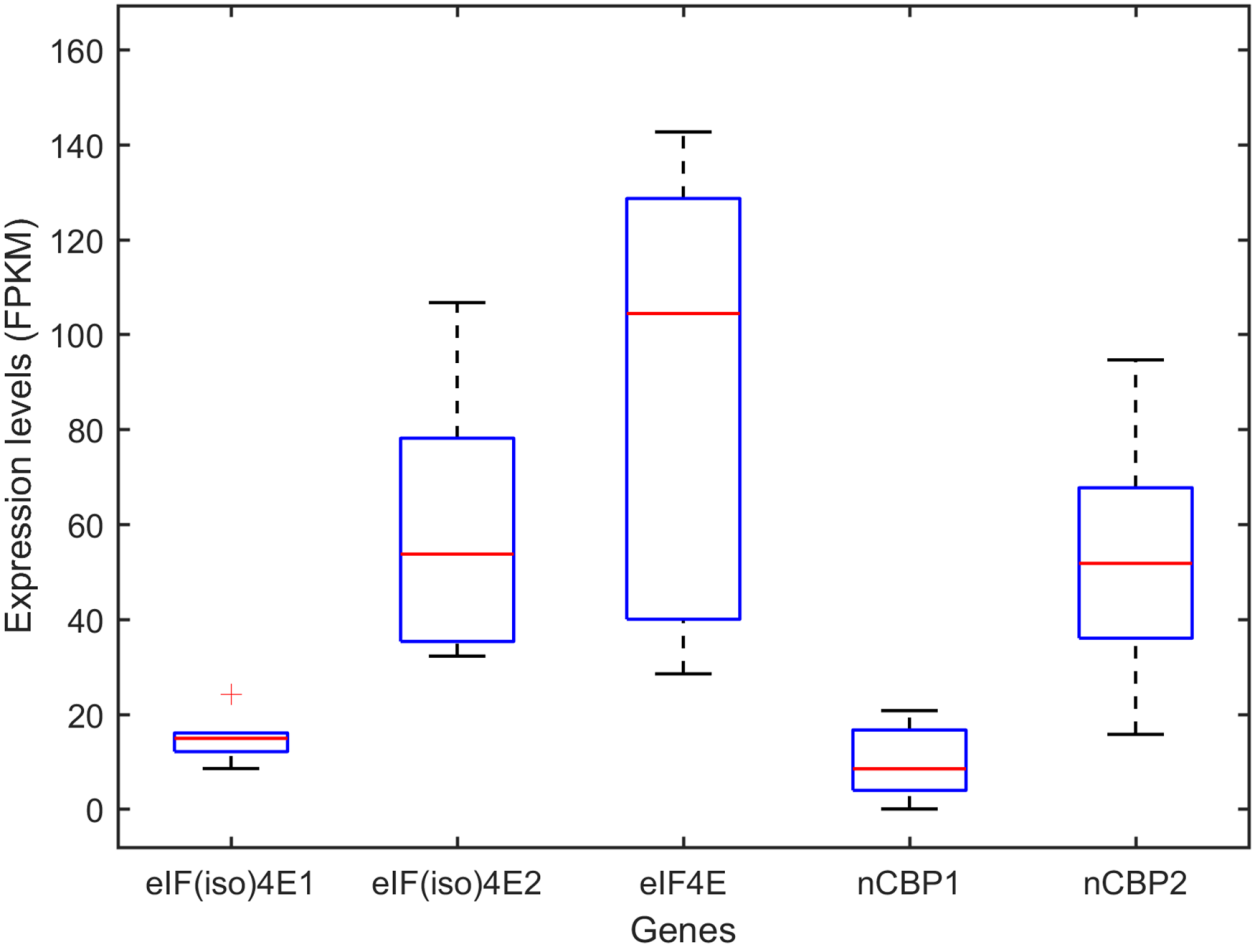
Boxplot of expression levels of eIF4E genes in six cassava lines. The 454 sequencing data were obtained from six cassava accessions (AR 37–80, AR40-6, Mkombozi, Kiroba, Nachinyaya, and Namikonga) [29]. Reads mapped to each gene were normalized to total read counts and lengths of each gene in kilobases (FPKM) and subsequently used as a measurement of expression levels. Statistical analyses and boxplotting were performed with MATLAB version R2017a.

**Fig 6:**
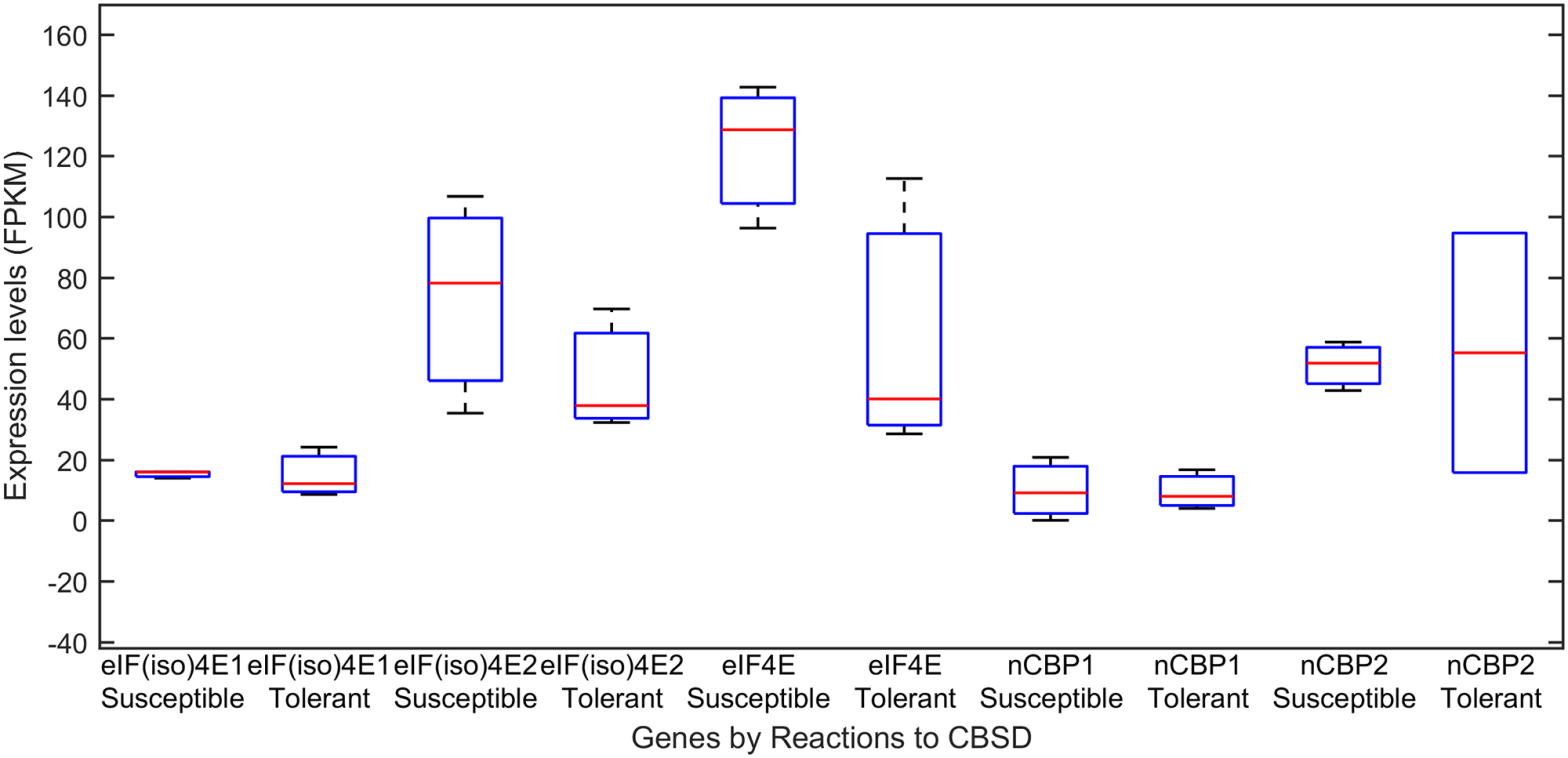
Boxplot of eIF4E gene expression levels of CDSD-susceptible and tolerant cassava accessions. The 454 sequencing data were obtained from three CBSD-susceptible accessions (AR 37–80, AR40-6, and Mkombozi) and three CBSD-tolerant accessions (Kiroba, Nachinyaya, and Namikonga) [29]. Reads mapped to each gene were normalized to total read counts and lengths of each gene in kilobases (FPKM) and subsequently used as a measurement of expression levels. Data from the susceptible and tolerant accessions were grouped together for statistical analysis. Each sample in the susceptible and tolerant group was considered as an independent sample. Statistical analyses and boxplotting were performed with MATLAB version R2017a.

Analyses of a second set of 454 Life Sciences sequencing data confirmed these results. This set of data were extracted from transcriptomic studies of CBSD-susceptible and CBSD-tolerant cassava accessions [29]. The original study contained 11 samples. However only data from three CBSD-susceptible accessions (AR 37–80, AR40-6, and Mkombozi) and three CBSD-tolerant accessions (Kiroba, Nachinyaya, and Namikonga) were used for this analysis. These data were selected using the criteria of total number of high quality sequencing reads and the percentage of reads mapped to the cassava genome (Table F in S1 File). While the total reads counts and reproducibility in house-keeping genes were generally low in this set of data (Figure D and Table F in S1 File), analyses of the expression of each eIF4E genes corroborated the significantly higher expression levels of cassava *eIF4E*, *eIF(iso)4E2*, and *nCBP2* genes than those of *eIF(iso)4E1* and *nCBP1* (Fig 5). When the gene expressions of the CBSD-susceptible and CBSD-tolerant cassava accessions were analyzed in groups, there was no significant difference in the expression level of each eIF4E genes (Fig 6), consistent with the results from the analysis of the Illuminate RNAseq data on Albert and Kaleso. However, there was a notable, but not statistically significant difference in the expression levels of the *eIF4E* gene between susceptible and tolerant cassava accession, with a higher level of expression in susceptible lines and a lower level of expression in tolerant lines (Fig 6). This observation is inconsistent with the result of *eIF4E* expression in the susceptible Albert and tolerant Kaleso with or without CBSV infections (Fig 4).

## Discussion

We reported here the cloning and analysis of the transcripts from five cassava eIF4E genes: *eIF4E_me*, *eIF(iso)4E_me1*, *eIF(iso)4E_me2*, *nCBP_me1*, and *nCBP_me2* (Fig 2). Data provided in this paper verified annotations from Cassava Genome V6.1 experimentally and provided additional data to refine the annotation of these five genes. *eIF4E*_me, *eIF(iso)4E_me2*, and *nCBP_me2* were expressed at a significantly higher level than *eIF(iso)4E_me1* and *nCBP_me1*, suggesting differential roles of these genes in protein translation initiation. Furthermore, we examined the genetic variations of these five cassava genes and surrounding regions across 14 cassava accessions and assessed their association with the tolerant and susceptible responses to CBSD, a devastating disease in cassava production in sub-Saharan Africa. Although a few SNPs are marginally associated with the disease response phenotypes, no definitive correlation has been established between any SNP and CBSD tolerance or susceptibility.

eIF4E family proteins consisted of eIF4E, eIF(iso)4E, and nCBP, a relatively new class of cap-binding proteins [47,48]. They are not only critical for cap-dependent translation of mRNA in eukaryotes, but also for the expression of (+)-sense, single-stranded RNA viral genomes with a covalently linked genome protein (VPg). The compatible interaction between eIF4E and VPg is a prerequisite for successful virus infection. For this reason, we examined eIF4Es in cassava and investigated their roles in the response of cassava to CBSD, a disease caused by two related but distinct potyviruses, CBSV and UCBSV.

The five eIF4E genes we cloned and sequenced are essentially as predicted from the annotated Cassava Genome V6.1 [32,33], indicating the high quality of the genome assembly and annotations. However, our results also showed a few discrepancies in two loci: Manes.15G044900 and Manes.09G140300. The 3’ UTR of two allelic *eIF(iso)4E_me2* transcripts were both shorter than the annotated transcript Manes.15G044900.1, suggesting additional, alternative polyadenylation sites for this gene. An *nCBP_me1a* transcript with a 5’ UTR longer than the annotated Manes.09G140300.1 transcript was also cloned, indicating an alternative transcription start site further upstream of the predicted one in Manes.09G140300. Two alternate transcripts were annotated at the Manes.17G063100 locus: Manes.17G063100.1 and Manes.17G063100.2, produced by alternate splicing events. Manes.17G063100.1 (eIF4E_me) was readily detected, but Manes.17G063100.2 of 146 nucleotides longer was undetected after screening 10 independent cDNA clones. It is conceivable that either Manes.17G063100.2 was a rare transcript or it was not actively transcribed in TMS60444. Our data suggest that experimental data are still an integral and indispensable part of accurate gene annotation despite rapid advances in theoretical predictions and annotation pipelines of genomic data.

Phylogenetic analysis clearly classified the five cassava eIF4E genes as one *eIF4E*, two *eIF(iso)4E*s, and two *nCBP*s (Fig 2). Duplications of eIF4E genes are not frequent in plants, found only in some plants such as soybean and maize [49]. Cassava encodes two members each of *eIF(iso)4E* and *nCBP*. The two eIF(iso)4E proteins are very similar, indicating a recent duplication event. However, the two nCBP proteins are quite divergent (Fig 2), indicating an earlier duplication event in the cassava genome. The divergent sequences might indicate functional diversification for the two proteins such as translating a subpopulation of mRNA species, or being differentially regulated spatially or temporally [1]. This functional diversification was further supported by the substitutions in the eight tryptophans highly conserved in the eIF4E family of proteins [1] (Figure B in S1 File). It is possible that some viruses could explore this diversification for translation of viral genomes with 5’ covalently linked proteins. Differential expression levels of the two copies of *eIF(iso)4E* and *nCBP*, however, suggests that only one copy plays a predominant role while the other copy might play a complementary role in normal cassava metabolism.

Natural and engineered mutations of single or few amino acids in alleles of eIF4E proteins have led to recessive resistance or immunity in plants against viruses with a genome-linked VPg [13,16,50] (Figure B in S1 File). We hypothesized that natural variations in the sequences of eIF4E protein might explain the differences in the responses of various cassava accessions to CBSD and examined the genetic variations across a selected group of cassava lines exhibiting tolerance or susceptibility to CBSD. The 14 cassava lines we selected for this study were phenotypically well characterized in their field responses to CBSD [22,29,42–44] and their genomes were also resequenced with a reduced representation genotyping-by-sequencing approach [32]. Analysis of the SNPs in the five cassava eIF4E genes confirmed the heterozygous nature of cassava, typical of outcrossing species, as well as the footprints of intensive cassava breeding programs. Biallelic variations were easily identifiable in all eIF4E genes in all the cassava lines (Figure A and Table 2 in S1 File). With the exception of AM560-2, all other cassava landraces and accessions contain at least two or more eIF4E genes with biallelic variations. Some accessions such as Albert, AR37-80, TME3, TME7, TMS30572, and TMS60444 contained at least three homozygous eIF4E genes, an indication of intense breeding effort. Our data showed that alleles of the same eIF4E gene inherited from different parents coexisted in some landraces. This information is particularly useful for future efforts in engineering eIF4E genes or in RNAi-mediated silencing for eIF4E genes for CBSD resistance as has been implemented against other potyviruses [51,52].

Even though there were a large number of SNPs in the eIF4E genes, most of them were located in the introns and noncoding regions of the exons as expected for highly conserved and critical genes. Only 51 non-synonymous SNPs were found in the eIF4E coding regions across 61 cassava accessions, and this was expected of any housekeeping gene under a high selection pressure. Most of the SNPs were contributed by the two wild type relative, the tree cassava and the rubber cassava [32]. Among the subset of 14 cassava accessions selected for the association study, only 13 non-synonymous SNP sites were informative. Due to the small population size (n = 14) and the small set of SNPs (n = 13), the results of this association study should be interpreted with caution. We did not find significant association between eIF4E SNPs and the responses of cassava lines to CBSD, however, two SNPs were found to be marginally associated with field CBSD responses with p-values between 0.03 and 0.08. All are higher than the adjusted threshold *p* value of 0.005 set for this study. In addition to the high *p* values, there was also no direct causal-effect relationship observed between any SNP and the disease responses. Cassava accessions with the same SNP exhibited both tolerant and susceptible responses (Table B in S1 File). For example, the association of L223H (A17G20187344T) in eIF4_me with the disease response phenotypes was best described by a dominant model with T dominant over A in association with the tolerance phenotype (Table 4). With the A/T heterozygotes and T/T homozygotes at this location, three of four accessions were tolerant but one accession was susceptible. Another example is K40E (C09_25948588T) in nCBP_me1. The overdominant model (Heterozygote advantage model) provided the probability of 0.08, but the C/T heterozygotes were found in four individuals in the tolerance phenotype but also in four individuals of the susceptibility phenotype (Table 4, Table B in S1 File). This is difficult to interpret biologically as the effect of a plant eIF4E protein to prevent or suppress virus replication is recessive in nature [13,16,50]. Based on this information, the weakly associated SNPs in the two eIF4E genes do not directly relate to the observed tolerance to CBSD and most likely mediate the effect as confounders at the best.

Interestingly, an expanded scan of SNPs in neighboring regions discovered a stronger association between disease responses and five SNPs in the intergenic regions and a gene upstream of *eIF4E_me* (Fig 3, Table C in S1 File). The *p* values of these associations were nearly one magnitude lower than those found in the eIF4E genes, and one of them was lower than the adjusted threshold value (*p* = 0.005) set for this study. Four of these SNPs were tightly clustered in the intergenic region within 10 nucleotides of each other, and about 0.5 kb upstream of *eIF4E_me*. These SNPs may likely affect transcription of this gene or the gene upstream. The gene upstream of *eIF4E_me* is a SRG1 homolog, initially identified as an upregulated *Arabidopsis* gene during senescence [46]. Subsequent reports [53–56] indicated that SRG1 was also upregulated during abiotic stress and infections by bacteria, fungi and viruses, in a manner similar to defense-related PR proteins. SRG1 carries two domains conserved in 2-oxoglutarate/Fe(II)-dependent dioxygenases and 2-oxoglutarate and Fe(II)-dependent oxygenase. A related protein, 1-aminocyclopropane-1-carboxylic acid (ACC) oxidase converted ACC to ethylene in the final step of ethylene biosynthesis [57], and ethylene was an important plant hormone regulating many aspects of plant defense responses against microbial infections [58]. It is highly likely that expression level of the SRP1 homolog in cassava is related to tolerant or susceptible responses to CBSD. Alternatively, the upstream region could affect the expression level of eIF4E_me, and subsequently the responses to CBSD. A larger population size is needed in future studies to further refine the association and to provide a more reliable estimate of the association between any SNP and the CBSD responses.

Digital expression profile analysis showed that only three eIF4E genes were highly expressed while one copy each of *eIF(iso)4E* and *nCBP* genes were poorly expressed (Figs 4 and 5), suggesting complementary and reserved roles of the duplicated copies of these eIF4E genes. Further analysis of the different expression between CBSD-susceptible and CBSD-tolerant cassava accessions revealed no significant difference in the expression of eIF4E genes. There was a notable, but statistically insignificant difference in the expression level of the *eIF4E_me* gene in the 454 data set (Fig 6), however this difference in the *eIF4E_me* was not found in the analysis of the dataset on Albert and Kaleso, a much better dataset (Fig 4). Additional analyses of more and higher quality of RNAseq data would be required to ascertain if the expression level of the *eIF4E* gene is different between CBSD-susceptible and CBSD-tolerant cassava accessions and if this difference could be attributed to the SNP upstream of the *eIF4E* gene that exhibited a weak association with CBSV responses.

Overall this study did not find any strong association between known SNPs in cassava eIF4E genes and cassava responses to CBSD. There is a strong possibility that the natural tolerance to CBSD and the causal viruses is due to restrictions of the tolerant cassava on infection processes other than translation initiation during viral infection. These processes include but are not limited to incompatibility at the viral replication level and at the translational level mediated by factors other than eIF4Es, viral cell-to-cell and systemic movement, and active defenses such RNAi and various forms of hypersensitive reactions ([15]. With the more affordable high-throughput sequencing and the increasing computation powers, it may be feasible to design and execute experiments than can discriminate factors contributing to cassava tolerance to CBSD.

## Supporting information

S1 File Supporting tables and figures listed below are provided in the file. **Table A. Distribution and characteristics of SNP sites in five eIF4E genes and neighboring regions in 61 cassava accessions**.
**Table B. Non-synonymous single nucleotide polymorphisms in five cassava eIF4E genes of 14 cassava accessions**.
**Table C. Association of SNPs upstream of eIF4E_me gene with CBSD disease responses**.
**Table D. Information on Illumina RNAseq unpaired reads of two cassava accessions and Bowtie mapping statistics**.
**Table E. Differential expression analysis of five cassava eIF4E genes in healthy and CBSV infected Albert and Kaleso accessions**.
**Table F. Information on 454 GS FLX Titanium RNAseq reads of six cassava accessions and Bowtie mapping statistics**.
**Figure A. Distribution of nucleotide polymorphisms in five cassava eIF4E genes across 10 landraces**. High quality Illumina reads of 10 cassava genomes are anchor-aligned to the cassava genome and viewed in IGV. Vertical colored lines indicate nucleotide polymorphisms. Each color represents a different nucleotide substitution. Black dots indicate deletions or missing data. Biallelic variations are clearly visible in certain genes of cassava lines.
**Figure B. Alignment of cassava eIF4E proteins with representative plant and human eIF4E proteins**. Proteins were aligned with ClustalX 2.0 and manually inspected and adjusted. Shading of similar amino acids and display were performed with BioEdit 7.09. Amino acids conserved in all eIF4E proteins are noted in the consensus line. Eight conserved tryptophan (W) amino acids are indicated with arrows and two involved in cap-binding are indicated in red arrows. Positions of eIF4E proteins where amino acid substitutions resulted in recessive resistance to potyviruses in nature or experimentally are indicated by red six-point stars. Protein sequences of additional eIF4E family proteins were retrieved from NCBI. Protein sources are identified with two letters representing the initials of genus and species, followed by NCBI accession number. At, *Arabidopsis thaliana*; Ca: *Capsicum annuum*; Cl, *Citrullus lanatus*; Cm, *Cucumis melo*; Cs, *Cucumis sativus*; Gm, *Glycine max*; Hs, *Homo sapiens*; Jc, *Jatropha curcas*; Ps, *Pisum sativum*; Pt, *Populus trichocarpal*; Pv, *Phaseolus vulgaris*; Rc, *Ricinus communis*; St, *Solanum tuberosum*; and Vv, *Vitis vinifera*.
**Figure C. Single nucleotide polymorphisms (SNPs) in the five cassava eIF4E genes and the upstream and downstream 5 kb regions**. Data were obtained from combined SNPs and indels from 61 cassava accessions curated at Phytozome (phytozome.jgi.doe.gov). Images of SNPs were rendered in the Genome Browser implemented at the same site. Blue diamonds: synonymous nucleotide substitutions in coding regions or substitutions in non-coding regions; yellow diamonds: non-synonymous substitutions in coding regions; red diamonds: missense substitutions; vertical green lines: indels. Only a 2kb region upstream of nCBP2 is available from the genomic sequence.
**Figure D. Expression levels of five eIF4E genes in CBSD-susceptible and -tolerant lines**. RNAseq long reads (0.5 kb) specific to each gene from each of the lines generated by the Life Sciences 454 sequencing were counted and normalized to total mapped reads from each sample to generate the FPKM counts. AR37-80, AR40-6, and Mkombozi were susceptible to CBSD while Kiroba, Nachinyaya, and Namikonga were tolerant to CBSD. Raw data were retrieved from NCBI SRA (accessions SRR955444-SRR955447, SRR955449, SRR955450, SRR955453, and SRR955456).
(PDF)
