# Supplementary material for: Variations of five eIF4E genes across cassava accessions exhibiting tolerant and susceptible responses to cassava brown streak disease
Source: PLoS One. 2017 Aug 3;12(8):e0181998. doi: 10.1371/journal.pone.0181998 (PMC5542559; doi:10.1371/journal.pone.0181998)
Supplement: S1 File — Table A. Distribution and characteristics of SNP sites in five eIF4E genes and neighboring regions in 61 cassava accessions. Table B. Non-synonymous single nucleotide polymorphisms in five cassava eIF4E genes of 14 cassava accessions. Table C. Association of SNPs upstream of eIF4E_me gene with CBSD disease responses. Table D. Information on Illumina RNAseq unpaired reads of two cassava accessions and Bowtie mapping statistics. Table E. Differential expression analysis of five cassava eIF4E genes in healthy and CBSV infected Albert and Kaleso accessions. Table F. Information on 454 GS FLX Titanium RNAseq reads of six cassava accessions and Bowtie mapping statistics. Figure A. Distribution of nucleotide polymorphisms in five cassava eIF4E genes across 10 landraces. High quality Illumina reads of 10 cassava genomes are anchor-aligned to the cassava genome and viewed in IGV. Vertical colored lines indicate nucleotide polymorphisms. Each color represents a different nucleotide substitution. Black dots indicate deletions or missing data. Biallelic variations are clearly visible in certain genes of cassava lines. Figure B. Alignment of cassava eIF4E proteins with representative plant and human eIF4E proteins. Proteins were aligned with ClustalX 2.0 and manually inspected and adjusted. Shading of similar amino acids and display were performed with BioEdit 7.09. Amino acids conserved in all eIF4E proteins are noted in the consensus line. Eight conserved tryptophan (W) amino acids are indicated with arrows and two involved in cap-binding are indicated in red arrows. Positions of eIF4E proteins where amino acid substitutions resulted in recessive resistance to potyviruses in nature or experimentally are indicated by red six-point stars. Protein sequences of additional eIF4E family proteins were retrieved from NCBI. Protein sources are identified with two letters representing the initials of genus and species, followed by NCBI accession number. At, Arabidopsis thaliana; Ca: Cap [file pone.0181998.s001.pdf]

Supporting information:

Table A. Distribution and characteristics of SNP sites in five eIF4E genes and neighboring regions in 61 cassava accessions

Loci*	Total SNP	Up/Downstream (+/- 5kb)	Genes	Introns	mRNA	CDS	Synonymous	Non-synonymous
eIF(iso)4E_me1	986	835	151	92	59	22	9	13
nCBP_me2	666	499	167	127	40	15	13	2
nCBP_me1	858	637	221	147	74	22	7	15
eIF(iso)4E_me2	992	780	212	175	37	13	9	4
eIF4E_me	886	776	110	68	42	30	13	17
Total	4388	3527	861	609	252	102	51	51

Table B. Non-synonymous single nucleotide polymorphisms in five cassava eIF4E genes of 14 cassava accessions

Chromosome	Position	AM560	Albert	AR37-80	Kibandameno*	Kiroba	MCOL22	Mkombozi	Muzege	Namikonga	Nachinyaya	TME3	TME7	TMS30572	TMS60444
Chr03	25396170	G/G	G/G	G/G	G/G	G/G	G/C	G/G	G/G	G/G	./.	G/G	G/G	G/G	G/G
Chr03	25396238	G/G	G/G	G/G	G/G	G/G	G/C	G/G	G/G	G/G	./.	G/G	G/G	G/C	G/C
Chr03	25396987	A/A	A/A	A/A	A/A	A/A	A/T	./.	A/A	A/A	./.	A/A	A/A	./.	A/T
Chr09	25948588	T/T	C/C	T/C	T/C	C/C	T/C	T/C	T/C	T/C	T/C	C/C	C/C	T/T	C/C
Chr15	3318172	C/C	C/T	C/T	./.	C/T	C/T	C/C	C/T	C/C	T/T	./.	C/C	C/C	C/C
Chr17	20187344	A/A	A/A	A/A	A/T	T/T	A/A	A/A	A/A	A/T	A/A	A/A	A/A	A/T	A/A
Chr17	20187753	T/T	T/T	T/T	T/T	T/C	T/T	T/T	T/T	T/T	T/T	T/T	T/T	T/T	T/T
Chr17	20188018	A/A	A/A	A/A	A/A	A/C	A/A	A/A	A/A	A/A	./.	A/A	A/A	A/A	A/A
Chr17	20188026	C/C	C/C	C/C	C/C	C/G	C/C	C/C	C/C	C/C	./.	C/C	C/C	C/C	C/C
Chr17	20189389	C/C	C/C	C/C	C/C	C/T	C/C	C/C	C/C	C/C	C/C	C/C	C/C	C/C	./.
Chr17	20189407	T/T	T/T	T/T	T/T	T/A	T/T	T/T	T/T	T/T	T/T	T/T	T/T	T/T	./.
Chr17	20189457	T/T	./.	T/T	T/T	T/C	T/T	T/T	T/T	T/T	T/T	./.	T/T	T/T	T/T
Chr17	20189544	T/T	./.	T/T	T/T	T/A	T/T	T/A	T/T	T/A	T/T	T/A	T/A	A/A	T/T

*Accessions in red display tolerant responses to CBSD while accessions in white display susceptible response to CBSD. Missing data are represented by "./."

Table C. Association of SNPs upstream of *eIF4E_me* gene with CBSD disease responses.

CHR	BP	SNP	P value	Regions	Substitutions
**Chr17**	20190229	ss.94871927	0.004	Intergenic	G/A
**Chr17**	20190238	ss.94871930	0.007	Intergenic	TA/T
**Chr17**	20190251	ss.94871935	0.007	Intergenic	A/G
**Chr17**	20190252	ss.94871936	0.007	Intergenic	TC/T
**Chr17**	20192085	ss.94872078	0.007	Manes.17G063200	G/T, T187K

Table D. Information on Illumina RNAseq unpaired reads of two cassava accessions and Bowtie mapping statistics[1].

								Reads mapped to genes					
**SRA accessions**	Cassava accessions	CBSV Infection	Tissues	Phenotypes	Bases	Total reads[2]	%mapped to genome	eIF4E	eIF(iso)4E1	eIF(iso)4E2	nCBP1	nCBP2	Actin
**SRR1213744**	Albert	Healthy	leaf	Susceptible	2628M	42768937	0.8765	1676	98	1041	351	4136	6497
**SRR1213745**	Albert	Infected	leaf	Susceptible	2921M	47596602	0.886	2203	154	1558	490	6171	8700
**SRR1213746**	Kaleso	Healthy	leaf	Tolerant	1894M	30730846	0.8634	1224	77	804	254	2744	3927
**SRR1213747**	Kaleso	Infected	leaf	Tolerant	2416M	47596602	0.886	2352	191	1536	422	2825	9677

[1]Raw sequencing reads were generated by Maruthi et al. in 2014 [1] and retrieved from http://www.ncbi.nlm.nih.gov/sra.

[2]Average read lengths are 51 bp.

Table E. Differential expression analysis of five cassava eIF4E genes in healthy and CBSV infected Albert and Kaleso accessions.

Genes	Cassava lines	Healthy (FPKM)	Infected (FPKM)	log2	test_stat	p[1]	q[2]
**eIF4E**	Albert	67.4627	80.5457	0.255717	0.447822	0.6613	0.998506
	Kaleso	68.9774	87.6125	0.345012	0.596169	0.54455	0.998506
**eIF(iso)4E1**	Albert	6.18663	8.75624	0.501157	0.599087	0.5463	0.998506
	Kaleso	6.74423	11.0195	0.708338	0.878567	0.36865	0.998506
**eIF(iso)4E2**	Albert	39.6478	53.6243	0.435647	0.752701	0.44975	0.998506
	Kaleso	42.7417	53.4709	0.32311	0.560435	0.5804	0.998506
**nCBP1**	Albert	9.56893	11.9476	0.320294	0.474463	0.6357	0.998506
	Kaleso	9.70205	10.443	0.106175	0.157073	0.87315	0.998506
**nCBP2**	Albert	135.035	181.42	0.426	0.720313	0.4565	0.998506
	Kaleso	125.056	83.9671	-0.574679	-0.990581	0.32255	0.998506

[1]The uncorrected p-value of the test statistic.

[2]The FDR-adjusted p-value of the test statistic. FDR is set at 0.05.

Table F. Information on 454 GS FLX Titanium RNAseq reads of six cassava accessions and Bowtie mapping statistics[1].

							Reads mapped to genes					
**SRA accessions**	Cassava accessions	Tissues	Phenotypes	Bases	Total reads[2]	%mapped to genome	eIF4E	eIF(iso)4E1	eIF(iso)4E2	nCBP1	nCBP2	Actin
**SRR955450**	AR37-80	leaf, stem	Susceptible	88M	190325	70.84%	19	2	5	8	4	32
**SRR955446**	AR40-6	leaf, stem	Susceptible	101M	208365	64.21%	17	2	15	7	0	68
**SRR955445**	Mkombozi	leaf, stem	Susceptible	168M	346000	66.89%	22	3	19	10	3	85
**SRR955449**	Kiroba	leaf, stem	Tolerant	123M	291436	60.83%	7	2	6	N/A	2	54
**SRR955447**	Nachinyaya	leaf, stem	Tolerant	141M	261139	68.06%	5	4	13	17	1	46
**SRR955444**	Namikonga	leaf, stem	Tolerant	99M	205405	61.29%	14	1	5	2	3	49

[1]Raw sequencing reads were generated by Ferguson et al in 2015 [2] and retrieved from http://www.ncbi.nlm.nih.gov/sra.

[2]Average read lengths are 500 bp.

A. eIF(iso)4E_me1 (Locus: Manes.03G160000)

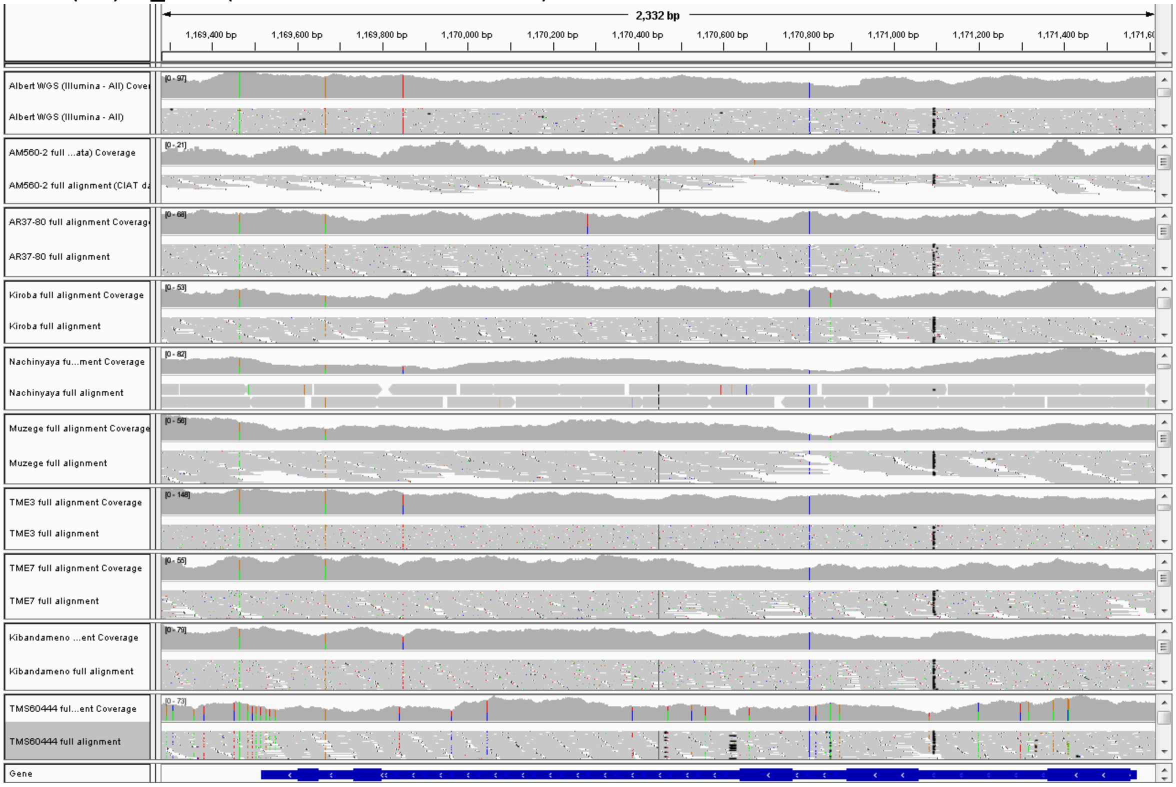

B. nCBP_me2 (Locus: Manes.08G145200)

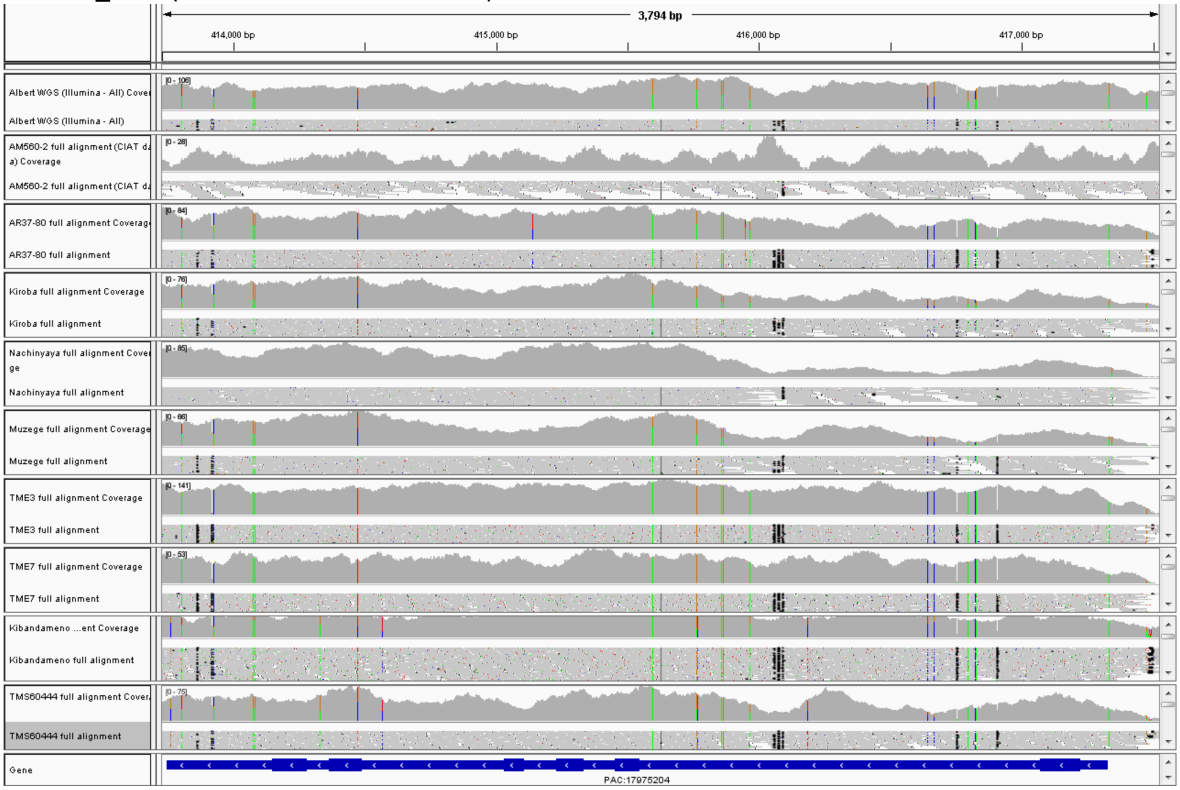

C. nCBP_me1 (Locus: Manes.09G140300)

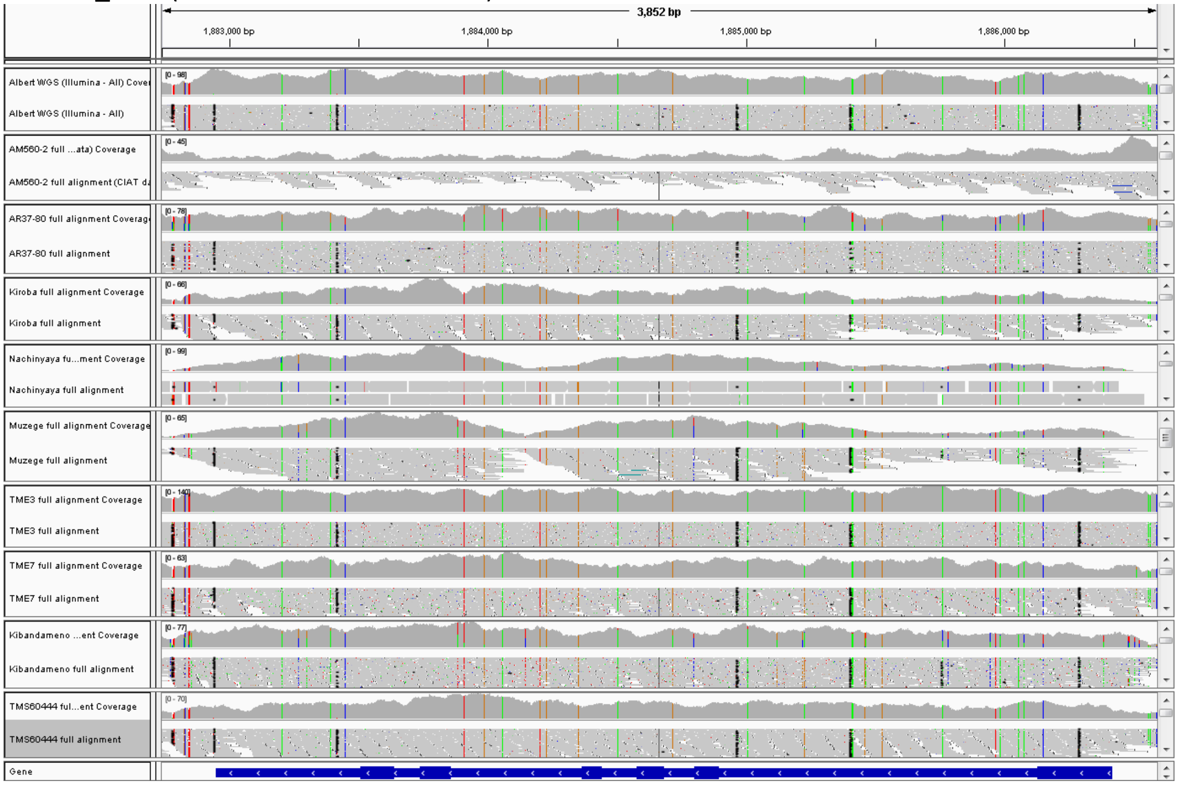

D. eIF(iso)4E_me2 (Locus: Manes.15G044900)

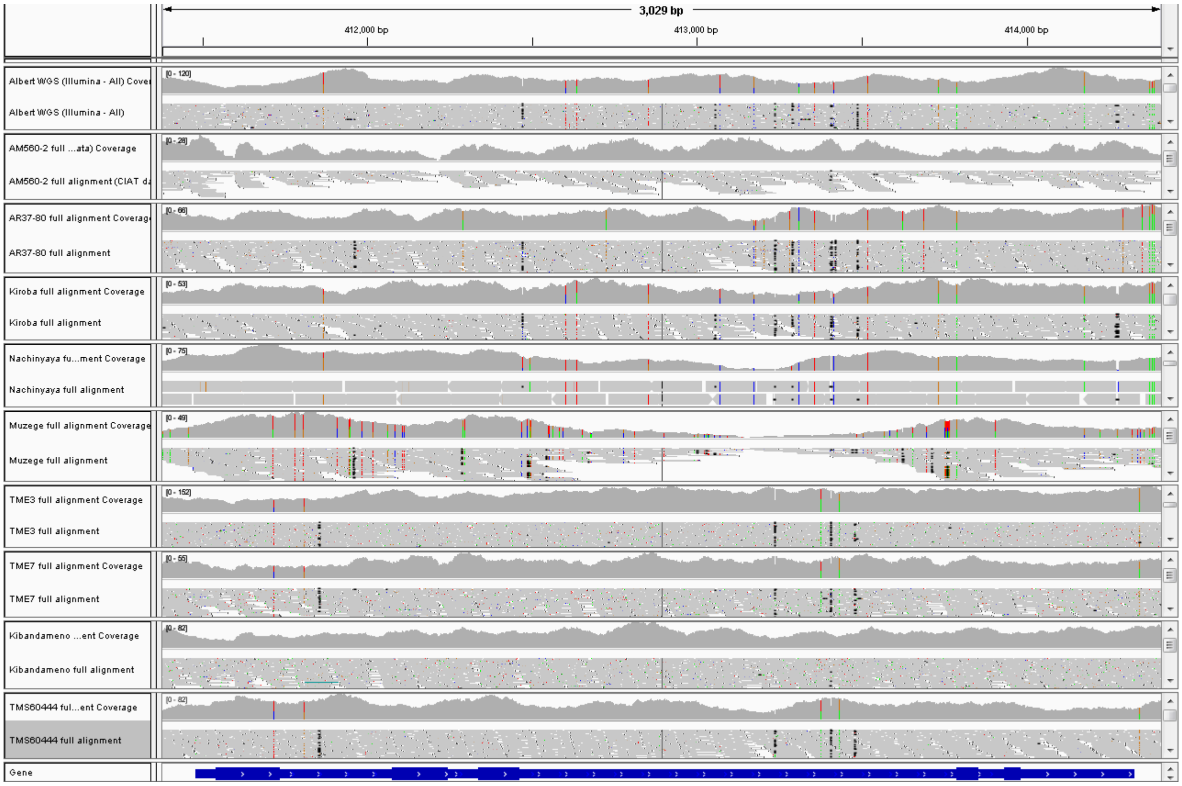

E. eIF4E_me (Locus: Manes.17G063100)

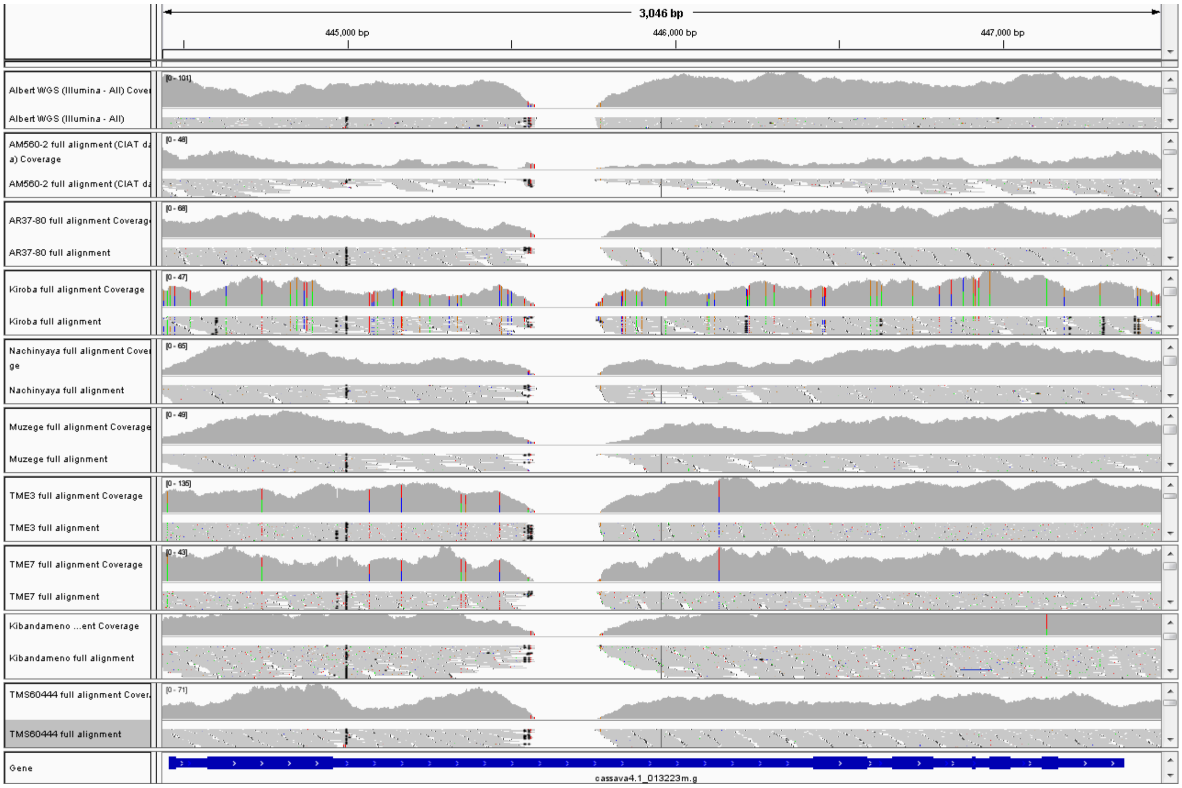

Figure A. Distribution of nucleotide polymorphisms in five cassava eIF4E genes across 10 landraces. High quality Illumina reads of 10 cassava genomes are anchor-aligned to the cassava genome and viewed in IGV [3]. Vertical colored lines indicate nucleotide polymorphisms. Each color represents a different nucleotide substitution. Black dots indicate deletions or missing data. Biallelic variations are clearly visible in certain genes of cassava lines.

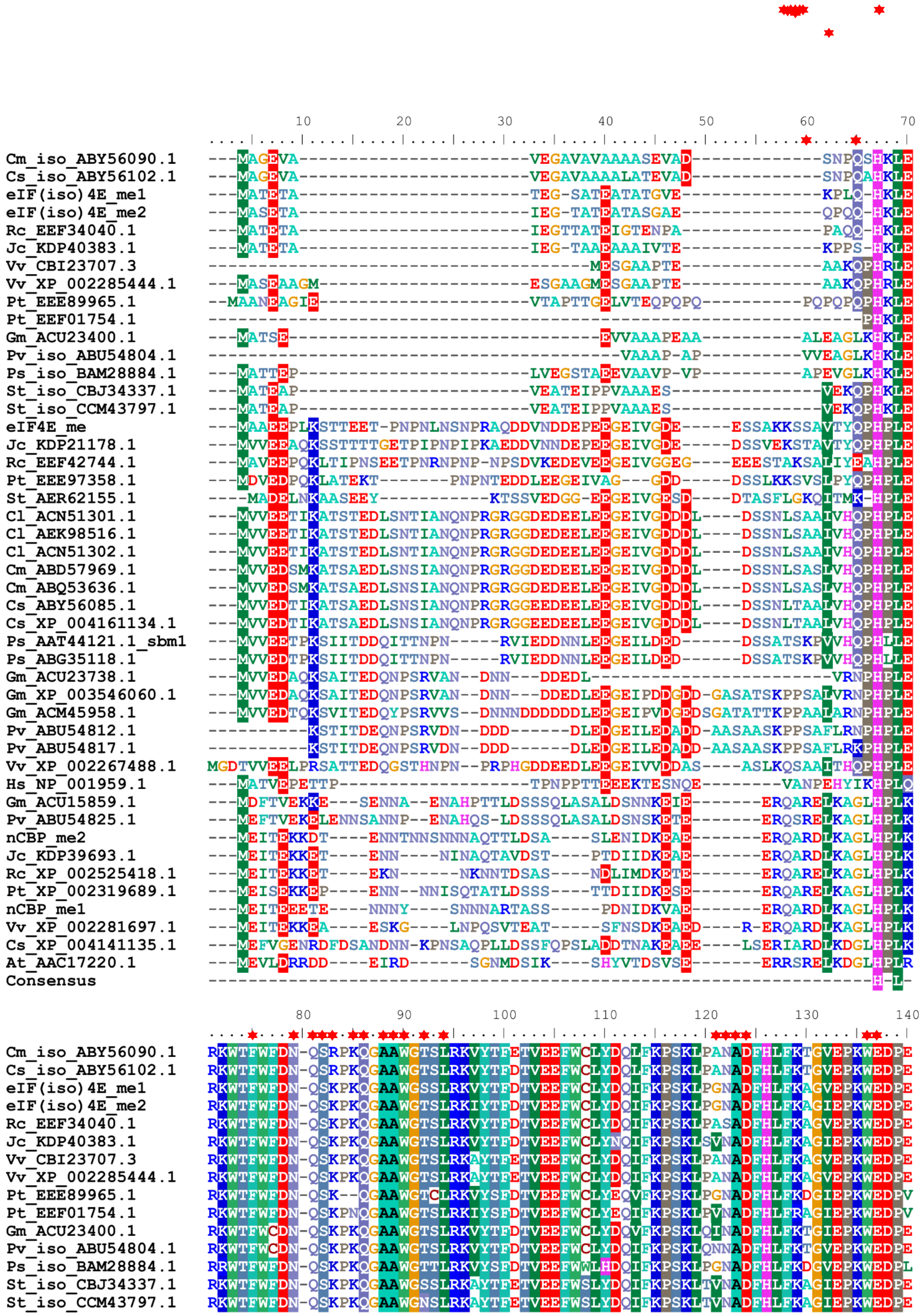
Cm_iso_ABY56090.1
Cs_iso_ABY56102.1
eIF(iso)4E_me1
eIF(iso)4E_me2
Rc_EEF34040.1
Jc_KDP40383.1
Vv_CBI23707.3
Vv_XP_002285444.1
Pt_EEE89965.1
Pt_EEF01754.1
Gm_ACU23400.1
Pv_iso_ABU54804.1
Ps_iso_BAM28884.1
St_iso_CBJ34337.1
St_iso_CCM43797.1
eIF4E_me
Jc_KDP21178.1
Rc_EEF42744.1
Pt_EEE97358.1
St_AER62155.1
Cl_ACN51301.1
Cl_AEK98516.1
Cl_ACN51302.1
Cm_ABD57969.1
Cm_ABQ53636.1
Cs_ABY56085.1
Cs_XP_004161134.1
Ps_AAT44121.1_sbm1
Ps_ABG35118.1
Gm_ACU23738.1
Gm_XP_003546060.1
Gm_ACM45958.1
Pv_ABU54812.1
Pv_ABU54817.1
Vv_XP_002267488.1
Hs_NP_001959.1
Gm_ACU15859.1
Pv_ABU54825.1
nCBP_me2
Jc_KDP39693.1
Rc_XP_002525418.1
Pt_XP_002319689.1
nCBP_me1
Vv_XP_002281697.1
Cs_XP_004141135.1
At_AAC17220.1
Consensus

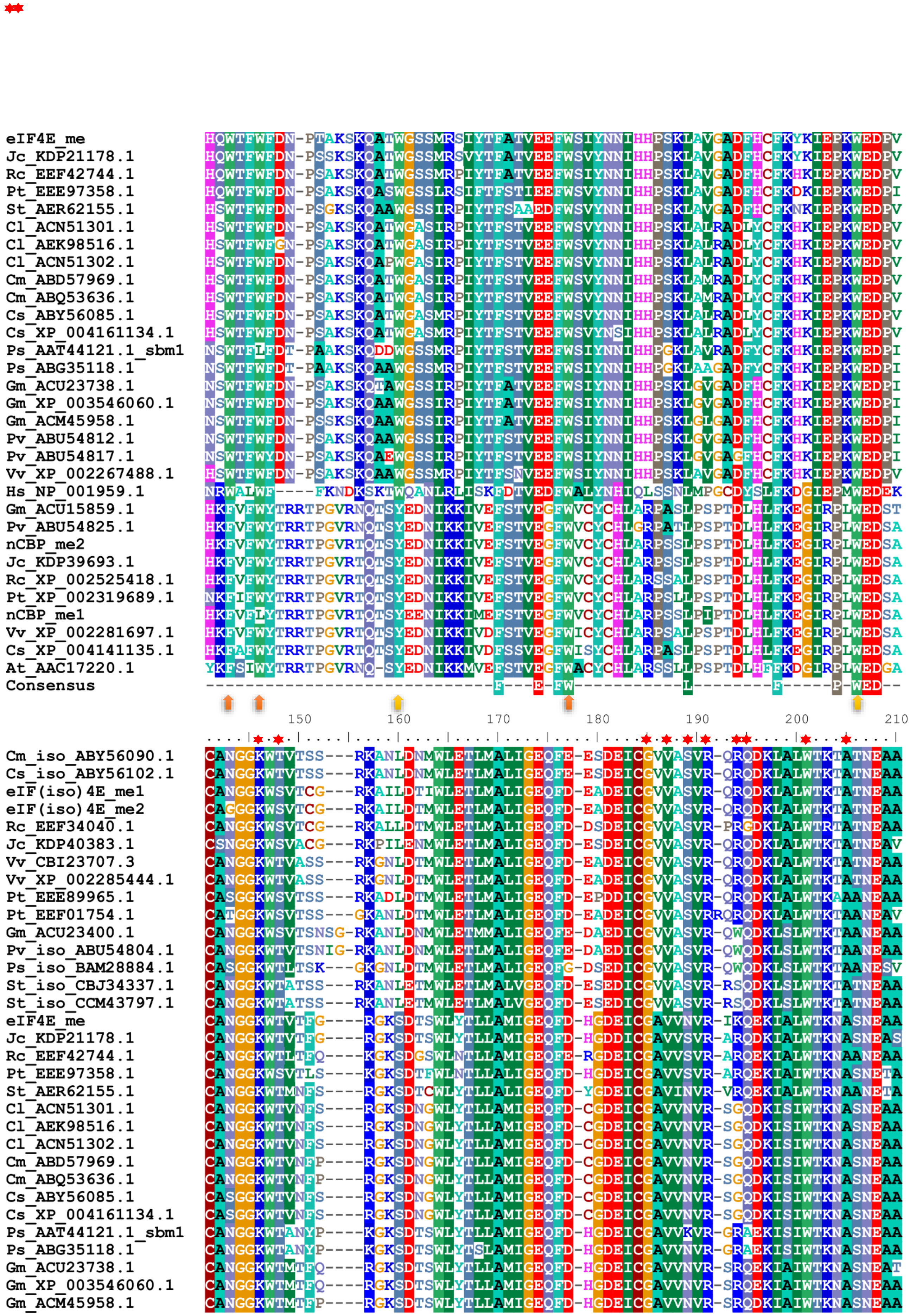

eIF4E_me
Jc_KDP21178.1
Rc_EEF42744.1
Pt_EEE97358.1
St_AER62155.1
Cl_ACN51301.1
Cl_AEK98516.1
Cl_ACN51302.1
Cm_ABD57969.1
Cm_ABQ53636.1
Cs_ABY56085.1
Cs_XP_004161134.1
Ps_AAT44121.1_sbm1
Ps_ABG35118.1
Gm_ACU23738.1
Gm_XP_003546060.1
Gm_ACM45958.1
Pv_ABU54812.1
Pv_ABU54817.1
Vv_XP_002267488.1
Hs_NP_001959.1
Gm_ACU15859.1
Pv_ABU54825.1
nCBP_me2
Jc_KDP39693.1
Rc_XP_002525418.1
Pt_XP_002319689.1
nCBP_me1
Vv_XP_002281697.1
Cs_XP_004141135.1
At_AAC17220.1
Consensus
150
160
170
180
190
200
210
Cm_iso_ABY56090.1
Cs_iso_ABY56102.1
eIF(iso)4E_me1
eIF(iso)4E_me2
Rc_EEF34040.1
Jc_KDP40383.1
Vv_CBI23707.3
Vv_XP_002285444.1
Pt_EEE89965.1
Pt_EEF01754.1
Gm_ACU23400.1
Pv_iso_ABU54804.1
Ps_iso_BAM28884.1
St_iso_CBJ34337.1
St_iso_CCM43797.1
eIF4E_me
Jc_KDP21178.1
Rc_EEF42744.1
Pt_EEE97358.1
St_AER62155.1
Cl_ACN51301.1
Cl_AEK98516.1
Cl_ACN51302.1
Cm_ABD57969.1
Cm_ABQ53636.1
Cs_ABY56085.1
Cs_XP_004161134.1
Ps_AAT44121.1_sbm1
Ps_ABG35118.1
Gm_ACU23738.1
Gm_XP_003546060.1
Gm_ACM45958.1

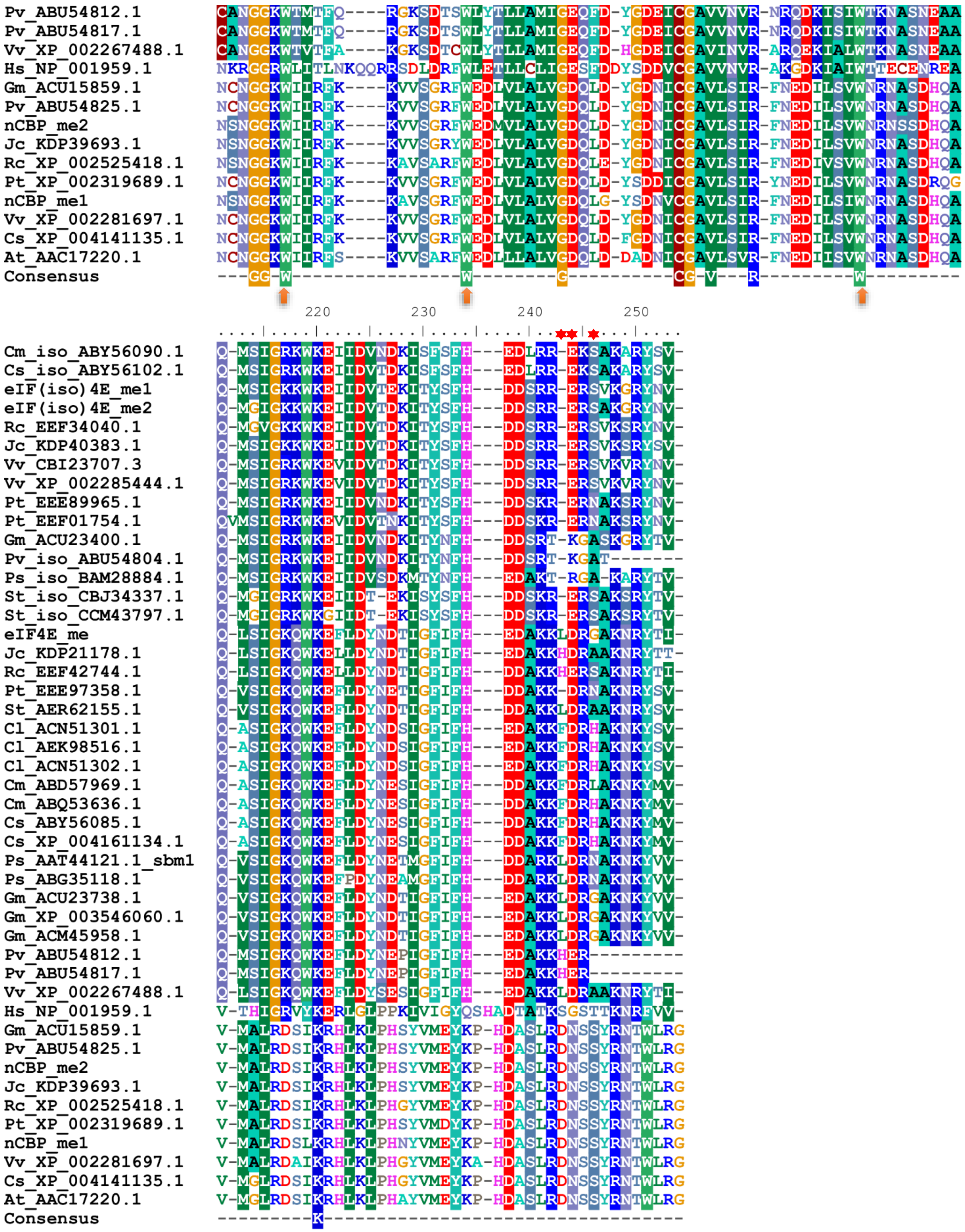
Pv_ABU54812.1
Pv_ABU54817.1
Vv_XP_002267488.1
Hs_NP_001959.1
Gm_ACU15859.1
Pv_ABU54825.1
nCBP_me2
Jc_KDP39693.1
Rc_XP_002525418.1
Pt_XP_002319689.1
nCBP_me1
Vv_XP_002281697.1
Cs_XP_004141135.1
At_AAC17220.1
Consensus
220
230
240
250
Cm_iso_ABY56090.1
Cs_iso_ABY56102.1
eIF(iso)4E_me1
eIF(iso)4E_me2
Rc_EEF34040.1
Jc_KDP40383.1
Vv_CBI23707.3
Vv_XP_002285444.1
Pt_EEE89965.1
Pt_EEF01754.1
Gm_ACU23400.1
Pv_iso_ABU54804.1
Ps_iso_BAM28884.1
St_iso_CBJ34337.1
St_iso_CCM43797.1
eIF4E_me
Jc_KDP21178.1
Rc_EEF42744.1
Pt_EEE97358.1
St_AER62155.1
Cl_ACN51301.1
Cl_AEK98516.1
Cl_ACN51302.1
Cm_ABD57969.1
Cm_ABQ53636.1
Cs_ABY56085.1
Cs_XP_004161134.1
Ps_AAT44121.1_sbm1
Ps_ABG35118.1
Gm_ACU23738.1
Gm_XP_003546060.1
Gm_ACM45958.1
Pv_ABU54812.1
Pv_ABU54817.1
Vv_XP_002267488.1
Hs_NP_001959.1
Gm_ACU15859.1
Pv_ABU54825.1
nCBP_me2
Jc_KDP39693.1
Rc_XP_002525418.1
Pt_XP_002319689.1
nCBP_me1
Vv_XP_002281697.1
Cs_XP_004141135.1
At_AAC17220.1
Consensus

Figure B. Alignment of cassava eIF4E proteins with representative plant and human eIF4E proteins. Proteins were aligned with ClustalX 2.0 [4] and manually inspected and adjusted. Shading of similar amino acids and display were performed with BioEdit 7.09 [5]. Amino acids conserved in all eIF4E proteins are noted in the consensus line. Eight conserved tryptophan (W) amino acids are indicated with arrows and two involved in cap-binding are indicated in red arrows. Positions of eIF4E proteins where amino acid substitutions resulted in recessive resistance to potyviruses in nature or experimentally [6,7] are indicated by red six-point stars. Protein sequences of additional eIF4E family proteins were retrieved from NCBI. Protein sources are identified with two letters representing the initials of genus and species, followed by NCBI accession number. At, *Arabidopsis thaliana*; Ca: *Capsicum annuum*; Cl, *Citrullus lanatus*; Cm, *Cucumis melo*; Cs, *Cucumis sativus*; Gm, *Glycine max*; Hs, *Homo sapiens*; Jc, *Jatropha curcas*; Ps, *Pisum sativum*; Pt, *Populus trichocarpal*; Pv, *Phaseolus vulgaris*; Rc, *Ricinus communis* ; St, *Solanum tuberosum*; and Vv, *Vitis vinifera*.

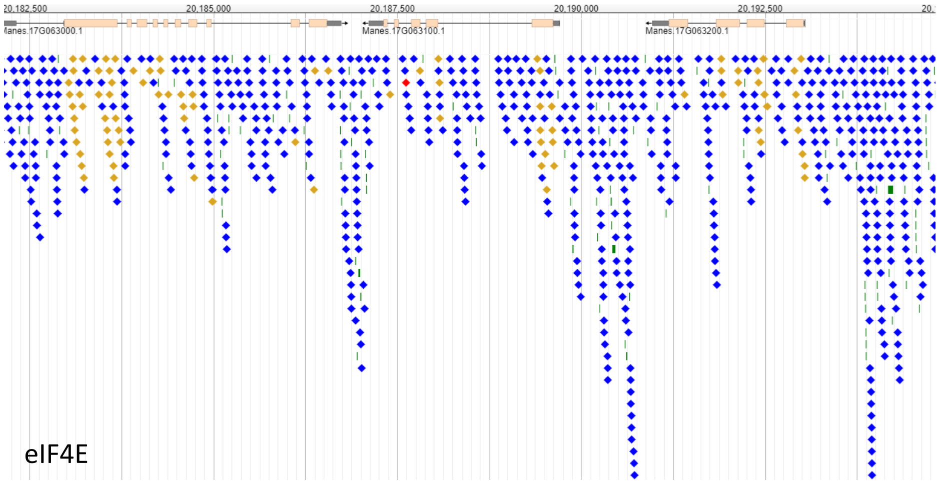
20,182,500
20,185,000
20,187,500
20,190,000
20,192,500
Manes.17G063000.1
Manes.17G063100.1
Manes.17G063200.1
eIF4E

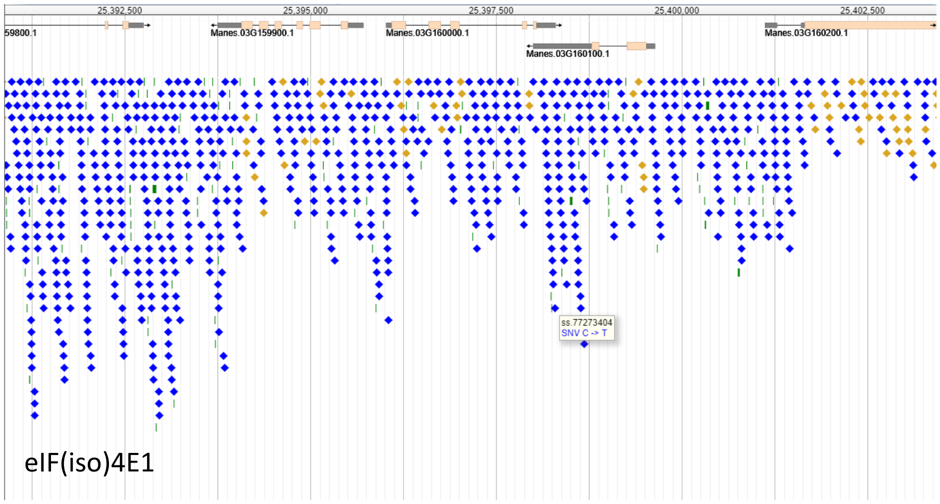
25,392,500
25,395,000
25,397,500
25,400,000
25,402,500
Manes.03G159900.1
Manes.03G160000.1
Manes.03G160100.1
Manes.03G160200.1
ss.77273404
SNV C -> T
eIF(iso)4E1

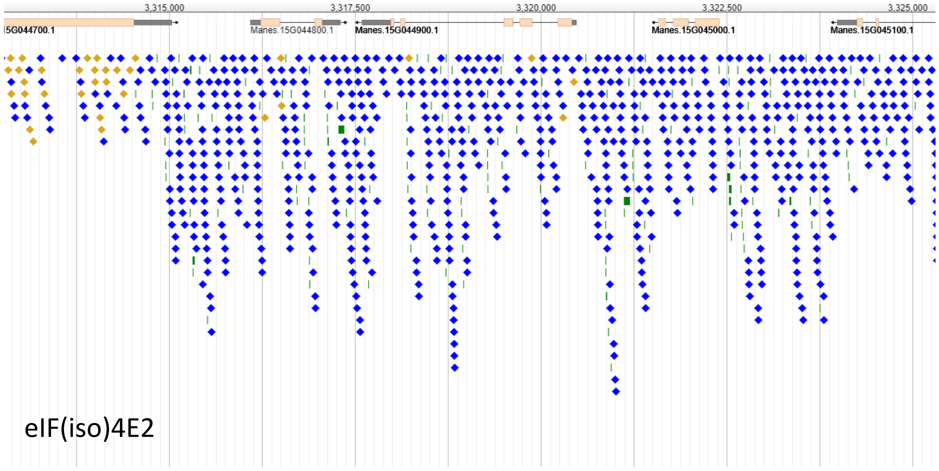
3,315,000
3,317,500
3,320,000
3,322,500
3,325,000
Manes.15G044800.1
Manes.15G044900.1
Manes.15G045000.1
Manes.15G045100.1
eIF(iso)4E2

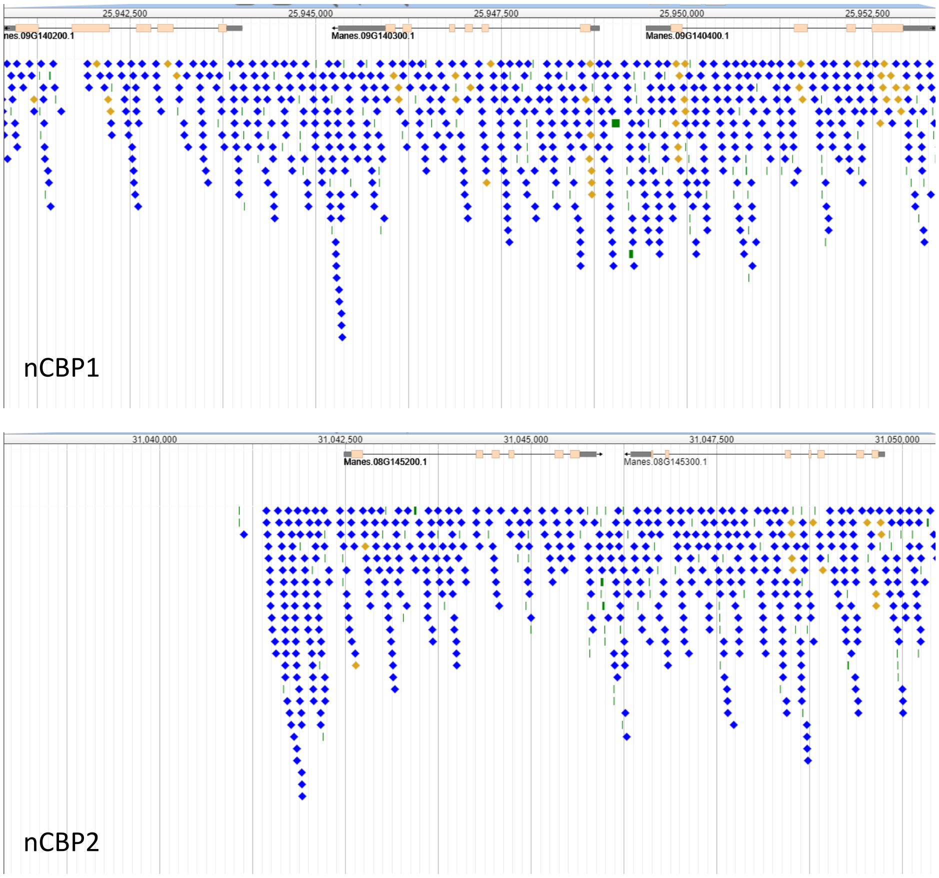

Figure C. Single nucleotide polymorphisms (SNPs) in the five cassava eIF4E genes and the upstream and downstream 5 kb regions. Data were obtained from combined SNPs and indels from 61 cassava accessions curated at Phytozome (phytozome.jgi.doe.gov). Images of SNPs were rendered in the Genome Browser implemented at the same site. Blue diamonds: synonymous nucleotide substitutions in coding regions or substitutions in non-coding regions; yellow diamonds: non-synonymous substitutions in coding regions; red diamonds: missense substitutions; vertical green lines: indels. Only a 2kb region upstream of nCBP2 is available from the genomic sequence.

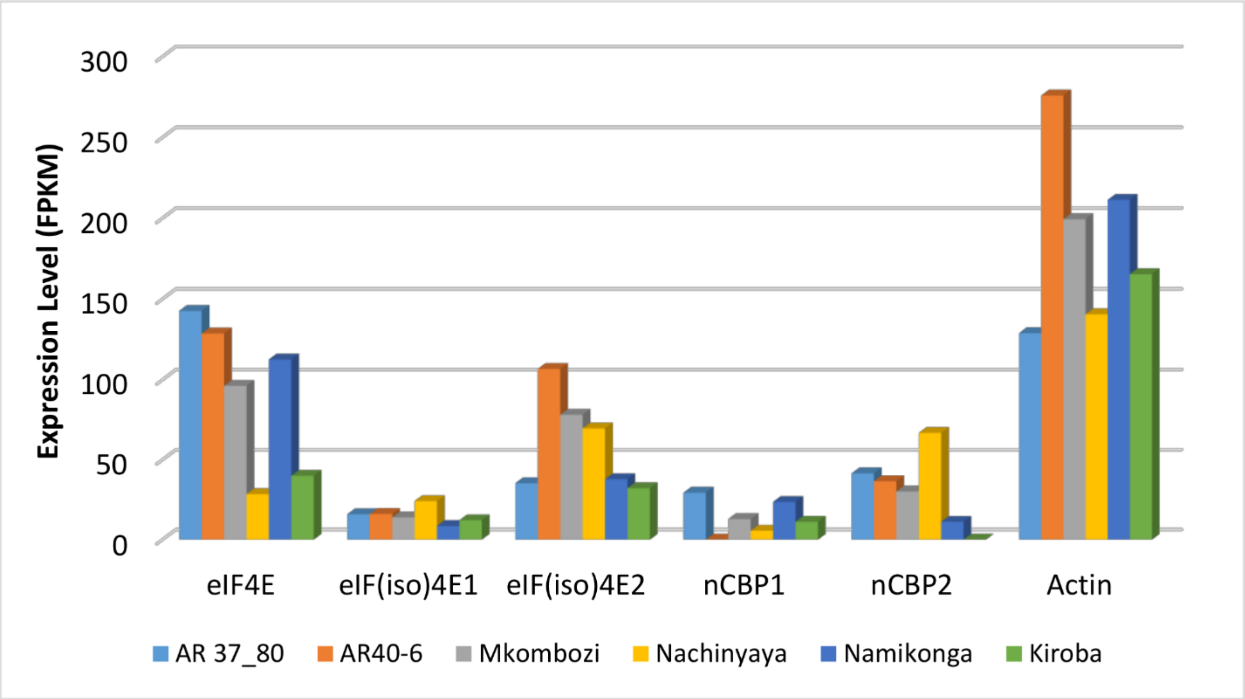

Figure D. Expression levels of five eIF4E genes in CBSD-susceptible and -tolerant lines. RNAseq long reads (0.5 kb) specific to each gene from each of the lines generated by the Life Sciences 454 sequencing were counted and normalized to total mapped reads from each sample to generate the FPKM counts. AR37-80, AR40-6, and Mkombozi were susceptible to CBSD while Kiroba, Nachinyaya, and Namikonga were tolerant to CBSD. Raw data were retrieved from NCBI SRA (accessions SRR955444-SRR955447, SRR955449, SRR955450, SRR955453, and SRR955456) [2].

References

1. Maruthi MN, Bouvaine S, Tufan HA, Mohammed IU, Hillocks RJ (2014) Transcriptional Response of Virus-Infected Cassava and Identification of Putative Sources of Resistance for Cassava Brown Streak Disease. Plos One 9.
2. Ferguson M, Koga T, Johnson D, Koga K, Hirsch G, et al. (2015) Identification of genes that have undergone adaptive evolution in cassava (Manihot esculenta) and that may confer resistance to cassava brown streak disease. African Journal of Biotechnology 14: 96-107.
3. Thorvaldsdottir H, Robinson JT, Mesirov JP (2013) Integrative Genomics Viewer (IGV): high-performance genomics data visualization and exploration. Briefings in Bioinformatics 14: 178-192.
4. Larkin MA, Blackshields G, Brown NP, Chenna R, McGettigan PA, et al. (2007) Clustal W and clustal X version 2.0. Bioinformatics 23: 2947-2948.
5. Hall TA. BioEdit: a user-friendly biological sequence alignment editor and analysis program for Windows 95/98/NT; 1999. [London]: Information Retrieval Ltd., c1979-c2000. pp. 95-98.
6. Ashby JA, Stevenson CEM, Jarvis GE, Lawson DM, Maule AJ (2011) Structure-Based Mutational Analysis of eIF4E in Relation to sbm1 Resistance to Pea Seed-Borne Mosaic Virus in Pea. Plos One 6.
7. Truniger V, Aranda MA (2009) Recessive Resistance to Plant Viruses. Advances in Virus Research, Vol 75 75: 119-+.
